# *O*‐GlcNAcylated LARP1 positively regulated by circCLNS1A facilitates hepatoblastoma progression through DKK4/β‐catenin signalling

**DOI:** 10.1002/ctm2.1239

**Published:** 2023-04-17

**Authors:** Zhongqi Cui, Jiangtu He, Jiabei Zhu, Wenxuan Ni, Li Liu, Zhixuan Bian, Siwei Mao, Song Gu, Yuhua Shan, Zhexuan Chu, Qi Wu, Jiayi Lu, Ya Liu, Fenyong Sun, Qiuhui Pan, Yue Zhang, Nan Huang, Ji Ma

**Affiliations:** ^1^ Department of Clinical Laboratory Shanghai Tenth People's Hospital, School of Medicine Tongji University Shanghai China; ^2^ Department of Clinical Laboratory Medicine Shanghai Children's Medical Center School of Medicine Shanghai Jiaotong University Shanghai China; ^3^ Department of Central Laboratory Clinical Medicine Scientific and Technical Innovation Park Shanghai Tenth People's Hospital Shanghai China; ^4^ Shanghai Key Laboratory of Clinical Molecular Diagnostics for Pediatrics Shanghai China; ^5^ Sanya Women and Children's Hospital Managed by Shanghai Children's Medical Center Sanya China; ^6^ Department of Surgery Shanghai Children's Medical Center School of medicine Shanghai Jiaotong University Shanghai China; ^7^ Department of Laboratory Medicine Hunan Children's Hospital Changsha China; ^8^ Shanghai Children's Hospital Shanghai Jiao Tong University Shanghai China; ^9^ Key Laboratory of Endemic and Ethnic Diseases, Ministry of Education Guizhou Medical University Guiyang China

**Keywords:** circCLNS1A, DKK4, hepatoblastoma, LARP1, *O*‐GlcNAcylation

## Abstract

**Background:**

Accumulating studies have shown that La‐related protein 1 (LARP1) is involved in the occurrence and development of various tumours. However, the expression pattern and biological role of LARP1 in hepatoblastoma (HB) remain unclear so far.

**Methods:**

LARP1 expression level in HB and adjacent normal liver tissues was analysed by qRT‐PCR, Western blotting and immunohistochemistry assays. The prognostic significance of LARP1 was evaluated by Kaplan–Meier method and multivariate Cox regression analysis. In vitro and in vivo functional assays were implemented to clarify the biological effects of LARP1 on HB cells. Mechanistically, the regulatory roles of *O*‐GlcNAcylation and circCLNS1A in LARP1 expression were investigated by co‐immunoprecipitation (co‐IP), immunofluorescence, RNA immunoprecipitation (RIP), RNA pull‐down and protein stability assays. Moreover, RNA‐sequencing, co‐IP, RIP, mRNA stability and poly(A)‐tail length assays were performed to investigate the association between LARP1 and DKK4. The expression and diagnostic significance of plasma DKK4 protein in multi‐centre cohorts were evaluated by ELISA and ROC curves.

**Results:**

LARP1 mRNA and protein levels were remarkably elevated in HB tissues and associated with worse prognosis of HB patients. LARP1 knockdown abolished cell proliferation, triggered cell apoptosis in vitro as well as prohibited tumour growth in vivo, whereas LARP1 overexpression incited HB progression. Mechanistically, *O*‐GlcNAcylation of LARP1 Ser672 by *O*‐GlcNAc transferase strengthened its binding to circCLNS1A and then protected LARP1 from TRIM‐25‐mediated ubiquitination and proteolysis. LARP1 upregulation subsequently led to DKK4 mRNA stabilisation by competitively interacting with PABPC1 to prevent DKK4 mRNA from B‐cell translocation gene 2‐dependent deadenylation and degradation, thus facilitating β‐catenin protein expression and nuclear import.

**Conclusion:**

This study indicates that upregulated protein level of *O*‐GlcNAcylated LARP1 mediated by circCLNS1A promotes the tumorigenesis and progression of HB through LARP1/DKK4/β‐catenin axis. Hence, LARP1 and DKK4 are promising therapeutical target and diagnostic/prognostic plasma biomarker for HB.

## Graphical abstract

- *O*‐GlcNAcylation of LARP1 Ser672 by OGT strengthened its binding to circCLNS1A and then protected LARP1 from TRIM‐25‐mediated ubiquitination and proteolysis.
- LARP1 upregulation subsequently led to DKK4 mRNA stabilisation by competitively interacting with PABPC1 to prevent DKK4 mRNA from BTG2‐dependent deadenylation and degradation.
- DKK4 promotes β‐catenin protein expression and nuclear induction to induce malignant progression of HB.

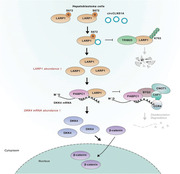

## 1 INTRODUCTION

Hepatoblastoma (HB) is a rare embryonal liver tumour that most commonly occurs in children younger than 5 years old.^1^, ^2^ Neoadjuvant chemotherapy and surgery lead to the survival of nearly 70% of patients with HB, particularly those with early‐stage disease.^3^, ^4^ However, detecting HB early remains challenging owing to its undetected onset, rapid progression and the absence of specific serum biomarkers.^5^ Patients with delayed diagnosis usually have poor prognosis owing to unresectable tumours and ineffective chemotherapy for advanced‐stage HB.^6^ To date, AFP is the only serum biomarker available for HB diagnosis given its elevation in approximately 90% of patients^7^; however, serum AFP level is also high in healthy infants and cannot distinguish HB from benign liver tumours and hepatocellular carcinoma (HCC).^8^, ^9^ Similarly, several immunohistochemical biomarkers, such as β‐catenin, GPC3 and INI1, cannot be used to confirm HB or HCC.^10^ Hence, it is an urgent need to identify the mechanisms underlying HB occurrence to develop biomarkers, identify targetable pathways and novel therapeutic avenues for patients with HB.

*O*‐Glycosylation modification can post‐translationally modify a variety of proteins by attaching *O*‐linked β‐*N*‐acetylglucosamine (*O*‐GlcNAc) to serine and threonine residues of proteins. The disruption of these modifications will alter the activity, stability and interactions of the target protein and play an important role in tumour development.^11^ Abnormal high levels of glycosylation modifications have been reported in various tumours, such as HCC, breast cancer and gastric cancer.^12^ Previous proteomic sequencing data showed that there was obvious disorder of glycosylation modification in HB,^13^ but the specific mechanism needs to be further explored.

La‐related proteins (LARPs) belong to a conserved family of RNA‐binding proteins that share the La‐motif and have specialised roles in RNA metabolism and protein synthesis.^14^ LARP1 interacts with RNA through its La‐motif and adjacent RNA‐recognition motif‐like (RRM‐L) domain, as well as the C‐terminal DM15 motif.^15^ LARP1 can stabilise mRNAs containing the 5′‐terminal oligopyrimidine (TOP) motif, which encodes translation initiation/elongation factors and ribosomal proteins.^16^ Moreover, post‐translational modification (PTM) of LARP1, here referring to phosphorylation, has been reported to determine its contrary regulatory role in TOP mRNA translation,^17^ and LARP1 has been connected to various cancers. It destabilises BIK mRNA but stabilises BCL2 mRNA via binary association with their 3′‐UTRs, maintaining ovarian cancer cell survival and chemotherapy resistance.^18^ LARP1 mRNA and protein levels are elevated in HCC tissues and are negatively associated with overall survival (OS) of early stage or AFP‐normal patients.^19^ Additionally, LARP1 phosphorylation by CDK2 enhances translation of oncogenic TOP‐ribosomal proteins and subsequent global translation, promoting HCC progression.^20^ However, LARP1's expression pattern and its pathological function in HB remain unknown.

In this study, we report a connection between LARP1 and *O*‐GlcNAc transferase (OGT), identify a novel oncogenic circCLNS1A/LARP1/DKK4 cascade, identify that DKK4 is a biomarker for HB diagnosis and prognosis and confirm that LARP1 is a promising therapeutic target.

## 2 MATERIALS AND METHODS

### 2.1 HB specimens collection

We collected 64 cases of HB and paracancerous tissues from Shanghai Children's Medical Center. All the subjects were the first diagnosed patients who did not receive any radiotherapy and chemotherapy. The clinicopathological information of patients is presented in Table S1. Plasma samples from patients with HB before surgery (*n* = 61), 1 day after surgery (*n* = 48), 3 days after surgery (*n* = 25) and 5 days after surgery (*n* = 20) were acquired from the Shanghai Children's Medical Center. Other plasma specimens were provided by healthy candidates (HC) (*n* = 101), and patients with chronic hepatitis B (*n* = 72), infantile hemangioendothelioma (IHE) (*n* = 38), HCC (*n* = 9) and HB (*n* = 126) at Shanghai Children's Medical Center and Shanghai Tenth people's hospital. Clinicopathological parameters from each specimen were analysed using multivariate Cox regression analysis to identify independent risk factors for OS and disease‐free survival (DFS). This study was approved by the Ethics Committee of the Shanghai Children's Medical Center and Shanghai Tenth People's Hospital.

### 2.2 Cell culture and treatment

The two human HB cell lines, HuH6 and HepG2, used in this study were obtained and cultured according to previous study.^21^

The *O*‐GlcNAcase inhibitor TMG and Proteasome inhibitors (MG132) were used at final concentrations of 10 μmol/L and 10 μM for 12 h, respectively. For mRNA stability assays, HB cells were incubated with 5 mg/mL actinomycin D at the indicated time points. To detect the lifetime of LARP1 protein, HB cells were incubated with 100 μg/mL cycloheximide (MedChemExpress) at the indicated time points. The detailed information for chemical reagents is presented in Table S2.

### 2.3 RNA pull‐down assay

A biotinylated circCLNS1A probe was synthesised by GenePharma Corporation, and the reverse probe was used as a control. RNA pull‐down assays were performed using the Pierce Magnetic RNA‐Protein Pull‐Down Kit (Thermo Fisher Scientific) according to the manufacturer's protocol. Briefly, 50 pmol of biotinylated circCLNS1A probe or reverse probe was incubated with 50 μL streptavidin‐coated magnetic beads for 30 min at room temperature. RNA‐beads were incubated with cell lysates at 4°C overnight. circCLNS1A enrichment was measured using qRT‐PCR. Proteins bound to the magnetic beads were analysed with SDS–PAGE to determine LARP1 levels.

### 2.4 Rapid amplification of cDNA ends‐poly(A) test (RACE‐PAT)

The poly(A)‐tail length of the DKK4 mRNA was measured using rapid amplification of cDNA ends‐poly(A) test (RACE‐PAT). Briefly, total RNA was reverse‐transcribed with an oligo(dT) primer linked to an oligo(dT) anchor. Next, PCR amplification was performed with the DKK4 forward primer for RACE and oligo(dT) anchor PCR primer, yielding a mixture of PCR‐amplified products representing the length of the DKK4 poly(A)‐tail. PCR products were resolved on a 5% non‐denaturing acrylamide gel. Primer sequences are listed in Table S8.

### 2.5 Xenograft tumour assay

Four‐week‐old male nude mice were purchased from Vital River Laboratory Animal Technology Corporation (Beijing, China). Each mouse was subcutaneously injected with 1 × 10^7^ HuH6 cells stably silencing/overexpressing LARP1 with or without infection with LV‐DKK4 or LV‐NC, or HuH6 cells infected with LV‐LARP1‐wild‐type (WT) or LV‐LARP1‐S672A. Each mouse received subcutaneous injections into the left (NC) and right (LARP1) flanks. Tumour length and width were measured every 4 days. Tumour volumes were calculated using the following formula: volume (mm^3^) = length (mm) × width^2^ (mm^2^) × .5. After 28 days, the mice were euthanised, and tumours were excised, weighed and imaged. IHC assays were conducted on the dissected tumours. The primary antibodies used were anti‐DKK4 (Proteintech, 27080‐1‐AP), anti‐Ki‐67 (Abcam, ab15580), anti‐PCNA (Abcam, ab18197) and anti‐β‐catenin (Abcam, ab16051).

### 2.6 HB orthotopic transplantation models

Briefly, 4‐week‐old male nude mice were anaesthetised with 10% chloral hydrate, and a longitudinal incision along the abdominal midline exposed the left lobe of the liver, where 1 × 10^8^ HepG2 cells infected with LV‐LARP1‐WT or LV‐LARP1‐S672A, suspended in 50 μL Matrigel, was injected using a 100 μL Hamilton syringe (Hamilton Company, Bonaduz, Switzerland). After suturing, the mice were caged individually and monitored until recovery. The survival period of HB in situ mice was analysed by Kaplan–Meier method using GraphPad Prism 7 (GraphPad Software, San Diego, CA, USA).

### 2.7 Detection of plasma AFP and DKK4

The concentrations of plasma AFP and DKK4 were measured using a Human AFP ELISA Kit (RayBiotech Life, Norcross, GA, USA) and Human Dkk‐4 (Dickkopf homolog‐4, ELISA Kit) (RayBiotech Life), respectively, using a Synergy 2 Multi‐Mode Microplate Reader (BioTek Instruments) in accordance with the manufacturer's instructions.

### 2.8 Detection of ubiquitination sites

We used the PhosphoSitePlus database (https://www.phosphosite.org/homeAction.action) to identify LARP1 ubiquitination sites. To determine whether ubiquitination occurred at these sites, we generated a panel of plasmids encoding point mutants of the LARP1 protein (Flag‐LARP1‐K539R/K703R/K753R) and then transfected them into sh‐circCLNS1A#1 cells.

### 2.9 Statistics

Data from at least three independent experiments are represented as mean ± SD and were analysed using GraphPad Prism 7.0 (GraphPad Software). Independent or paired T test is used to evaluate the significance of differences between the two groups’ comparisons, and one‐way ANOVA is used to evaluate the significance of differences among multiple groups’ comparisons. Pearson's *χ* ^2^ tests were used to analyse LARP1 protein expression in HB tissues. The effectiveness of DKK4 and AFP in diagnosing HB is analysed by ROC curve. OS and DFS curves were plotted to estimate survival using the Kaplan–Meier method, and the log‐rank test was applied for comparison. *p Value < .05, **p value < .01 or ***p value < .001 were considered statistically significant.

## 3 RESULTS

### 3.1 Significant upregulation of LARP1 in HB tissues correlates with poor prognosis of HB patients

**FIGURE 1:**
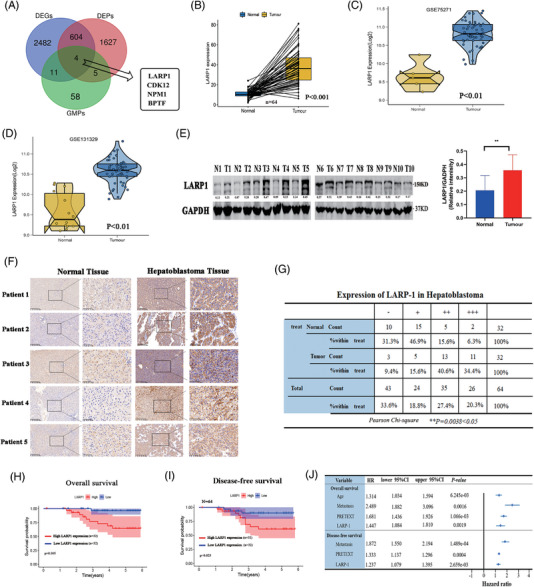
La‐related protein 1 (LARP1) is upregulated in hepatoblastoma (HB) tissues and correlates with worse prognosis of HB patients. (A) Venn diagram showed intersectional targets among differentially expressed genes (DEGs), differentially expressed proteins (DEPs) and GMPs in four paired HB and normal tissues. (B) LARP1 mRNA level in our collection of 64 HB tissue specimens was detected by qRT‐PCR. (C and D) LARP1 mRNA level in normal (*N*) and HB (*T*) tissues from two Gene Expression Omnibus (GEO) mRNA expression profiling datasets (GSE75271: *N* = 5, *T* = 50; GSE131329: *N* = 14, *T* = 53). (E) LARP1 protein level in 10 paired HB and normal tissues was detected by Western blotting assay. (F) Representative IHC images of LARP1 staining in 32 paired HB and normal tissues. (G) Pearson *χ* ^2^ test was used to analyse LARP1 protein expression in HB tissues. (H and I) Overall survival (OS) (H) and disease‐free survival (DFS) (I) analyses of 64 HB patients with high or low LARP1 mRNA expression (defined by the median 2^−ΔΔ^ *^Ct^* value of LARP1 expression in tissues). (J) Multivariate Cox regression analyses of clinicopathological characteristics for OS and DFS of HB patients.

We previously conducted combined analyses of transcriptomics, proteomics and *O*‐GlcNAcomics in four paired HB and adjacent normal liver tissues.^22^ In this study, four common genes (LARP1, CDK12, NPM1 and BPTF) between the differentially expressed genes, proteins and *O*‐GlcNAc modifications (GMPs) were obtained using a Venn diagram (Figure 1A). We verified the expression of four shared genes in 64 HB tissues and normal tissues. qRT‐PCR results showed the most significant differential expression of LARP1 (Figure 1B and Figure S1A). Two mRNA expression profiling datasets from the Gene Expression Omnibus (GEO) database also indicated increased LARP1 mRNA levels in HB tissues compared to normal tissues (Figure 1C,D). Western blotting assays confirmed that LARP1 expression in HB tissues was significantly higher than those in the adjacent normal tissues (Figure 1E). In IHC analysis of a tissue microarray, most HB tissues exhibited moderate (++) (40.6%) or strong (+++) (34.4%) positive staining for LARP1 compared with adjacent normal tissues (Figure 1F,G and Figure S1B). According to the median value of LARP1 mRNA expression level, 64 patients were divided into high and low expression groups. The Kaplan–Meier survival curve analysis revealed that patients with high LARP1 levels had worse OS and DFS than those with low levels (Figure 1H,I). Multivariate Cox regression analysis revealed that age, metastasis, pre‐treatment extent of tumour (PRETEXT) and LARP1 mRNA levels were independent prognostic indicators of OS. Furthermore, metastasis, PRETEXT and LARP1 mRNA levels were independent predictive factors for DFS (Figure 1J). These data suggest that LARP1 is upregulated in HB tissues and may be prognostic for patients with HB.

### 3.2 LARP1 stimulates HB tumour growth and represses apoptosis

**FIGURE 2:**
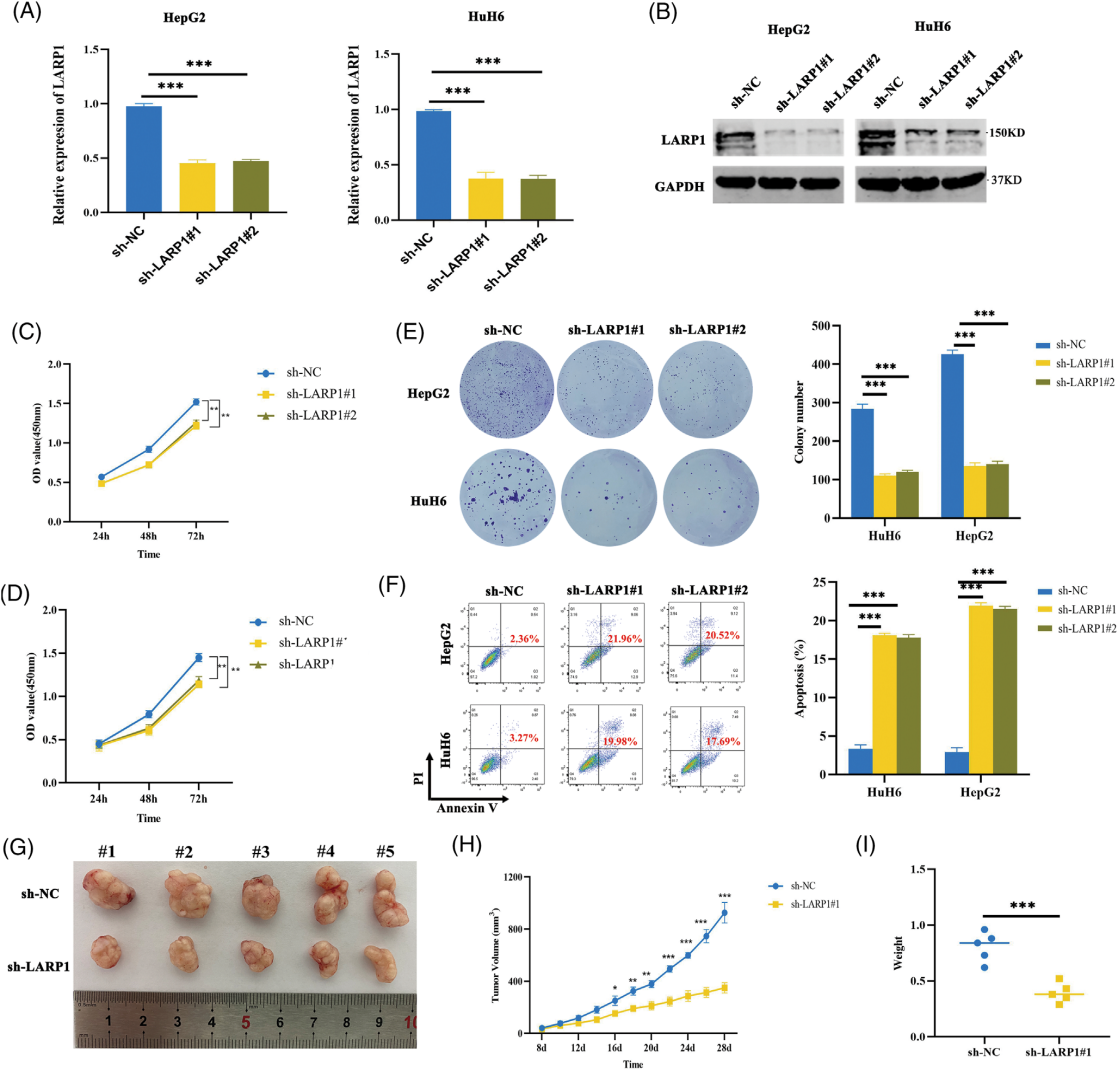
La‐related protein 1 (LARP1) knock‐down inhibits hepatoblastoma (HB) cell proliferation and promotes cell apoptosis. (A and B) LARP1 mRNA (A) and protein (B) levels in HB/sh‐NC and HB/sh‐LARP1#1, #2 cells were detected by qRT‐PCR and Western blotting assays. (C–E) The proliferative activities of HB/sh‐NC and HB/sh‐LARP1#1, #2 cells were examined using CCK‐8 (C and D) and colony formation (E) experiments. (F) Cell apoptosis analyses of HB/sh‐NC and HB/sh‐LARP1#1, #2 cells by flow cytometry assays. (G–I) The stripped tumours from five nude mice subcutaneously implanted with HuH6/sh‐NC and HuH6/sh‐LARP1#1 cells. (H) Tumour growth curve was depicted by calculating tumour volumes on indicated days. (I) Average weight of stripped tumours. *p < .05, **p < .01 and ***p < .001 between indicated groups.

To clarify the role of LARP1 in HB, we stably knocked down LARP1 in HB cells with a lentiviral shRNA. qRT‐PCR and Western blotting validated the reduced mRNA and protein levels of LARP1 in sh‐LARP1#1 and #2 cells, respectively (Figure 2A,B). The knock‐down of LARP1 significantly inhibited the growth and colony formation of HB cells (Figure 2C–E). Flow cytometry analysis confirmed that LARP1 depletion enhanced apoptosis (Figure 2F). In vivo xenograft studies indicated that the sh‐LARP1#1 group's tumour had lower volume and weight than those in the sh‐NC group (Figure 2G–I). Additionally, LARP1 overexpression significantly increased cell proliferation in vitro (Figure S1C–E) and promoted tumour growth in vivo (Figure S1F–H). Collectively, these results indicate that LARP1 plays an oncogenic role in HB.

### 3.3 Oncogenic effects of LARP1 on HB cells are mediated by upregulation of DKK4 expression

**FIGURE 3:**
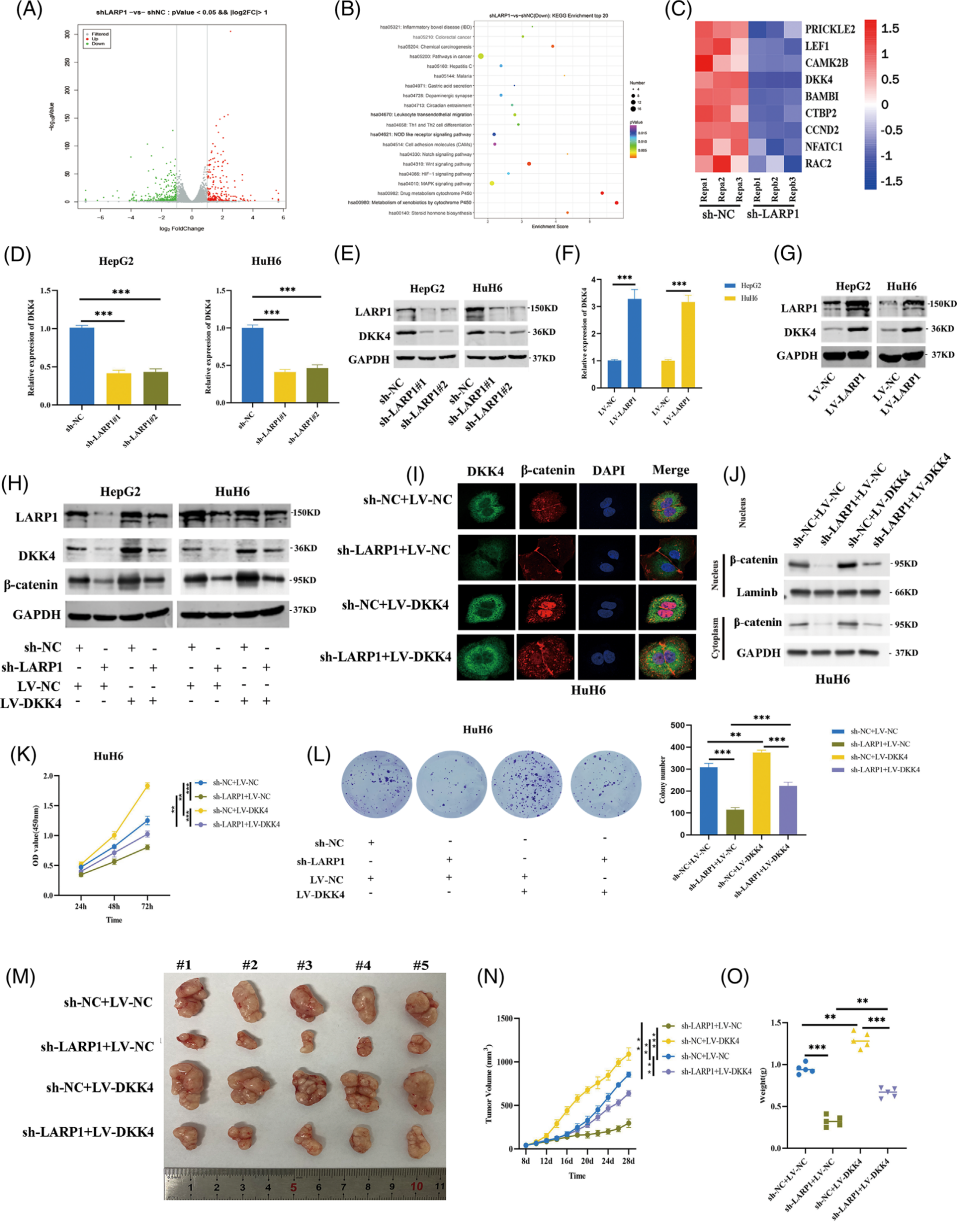
La‐related protein 1 (LARP1) upregulates DKK4 expression level to exert oncogenic roles in hepatoblastoma (HB). (A and B) Volcano plot (A) and Kyoto Encyclopedia of Genes and Genomes (KEGG) analysis (B) of 596 differentially expressed genes between HuH6/sh‐NC and HuH6/sh‐LARP1#1 cells detected by RNA‐seq. (C) Heat map exhibited downregulation of 9 Wnt‐related genes in HuH6/sh‐LARP1#1 cells versus HuH6/sh‐NC cells. (D–G) DKK4 mRNA and protein levels in HB/sh‐NC and HB/sh‐LARP1#1, #2 cells (D and E) or HB/LV‐NC and HB/LV‐LARP1 cells (F and G) were detected by qRT‐PCR and Western blotting assays. (H) Western blotting analyses for LARP1, DKK4 and β‐catenin protein levels in HB/sh‐NC and HB/sh‐LARP1#1 cells co‐infected with LV‐NC or LV‐DKK4. (I) HuH6/sh‐NC and HuH6/sh‐LARP1#1 cells co‐infected with LV‐NC or LV‐DKK4 were adopted for IF assays to detect the expression and localisation of DKK4 and β‐catenin. (J) Western blotting analyses for β‐catenin protein level in nuclear and cytoplasmic fractions of HuH6/sh‐NC and HuH6/sh‐LARP1#1 cells co‐infected with LV‐NC or LV‐DKK4. (K and L) The proliferative activities of HuH6/sh‐NC and HuH6/sh‐LARP1#1 cells co‐infected with LV‐NC or LV‐DKK4 were examined using CCK‐8 (K) and colony formation (L) experiments. (M–O) The stripped tumours from five nude mice subcutaneously implanted with HuH6/sh‐NC and HuH6/sh‐LARP1#1 cells co‐infected with LV‐NC or LV‐DKK4. (N) Tumour growth curve was depicted by calculating tumour volumes on indicated days. (O) Average weight of stripped tumours. **p < .01 and ***p < .001 between indicated groups.

To explore the downstream mechanisms of LARP1, HuH6 cells were stably silenced for LARP1. RNA sequencing revealed that there were 596 aberrantly expressed genes (341 downregulated genes and 255 upregulated genes) compared with sh‐NC cells (Figure 3A and Table S2). The Kyoto Encyclopedia of Genes and Genomes pathway enrichment analysis revealed that the Wnt signalling pathway related to HB progression was enriched and ranked among the top 20 signalling pathways (Figure 3B). Unsupervised hierarchical clustering revealed nine downregulated genes involved in the Wnt signalling pathway, of which DKK4 ranked first (Figure 3C). qRT‐PCR and Western blotting assays verified that DKK4 mRNA and protein levels were diminished in sh‐LARP1 cells compared to sh‐NC cells (Figure 3D,E) but increased with LARP1 overexpression (Figure 3F,G). To further probe the role of DKK4, rescue assays were performed by overexpressing DKK4 in HB cells stably silenced LARP1. Western blotting and IF assays showed that increased DKK4 levels partially rescued decreased β‐catenin protein levels and its nuclear translocation (Figure 3H,I and Figure S2A). Consistently, nucleo‐cytoplasmic separation assays also confirmed this phenomenon (Figure 3J and Figure S2B). Moreover, DKK4 overexpression partially rescued tumour proliferative and anti‐apoptotic effects (Figure 3K–O and Figure S2C–E). Considering that LARP1 can regulate DKK4 expression in HB cells, we further explored the relationship between LARP1 and DKK4 expression in HB tissues. Both GEO datasets and qRT‐PCR analyses indicated a remarkable elevation in DKK4 mRNA levels in HB tissues compared with normal tissues (Figure S3A–C). Pearson's correlation analysis revealed a positive correlation between LARP1 and DKK4 mRNA levels in HB tissues (*n* = 41) (Figure S3D). Consistently, IHC analysis also revealed a positive correlation between LARP1 and DKK4 protein expression in HB tissues (Spear‐man correlation *r* = .52, *p* = .013) (Figure S3E,F). Thus, these data imply that LARP1 activates the Wnt/β‐catenin signalling pathway to promote the malignant phenotype of HB cells by upregulating DKK4 expression.

### 3.4 LARP1 enhances DKK4 transcript stability by competing with BTG2 for binding to PABPC1

**FIGURE 4:**
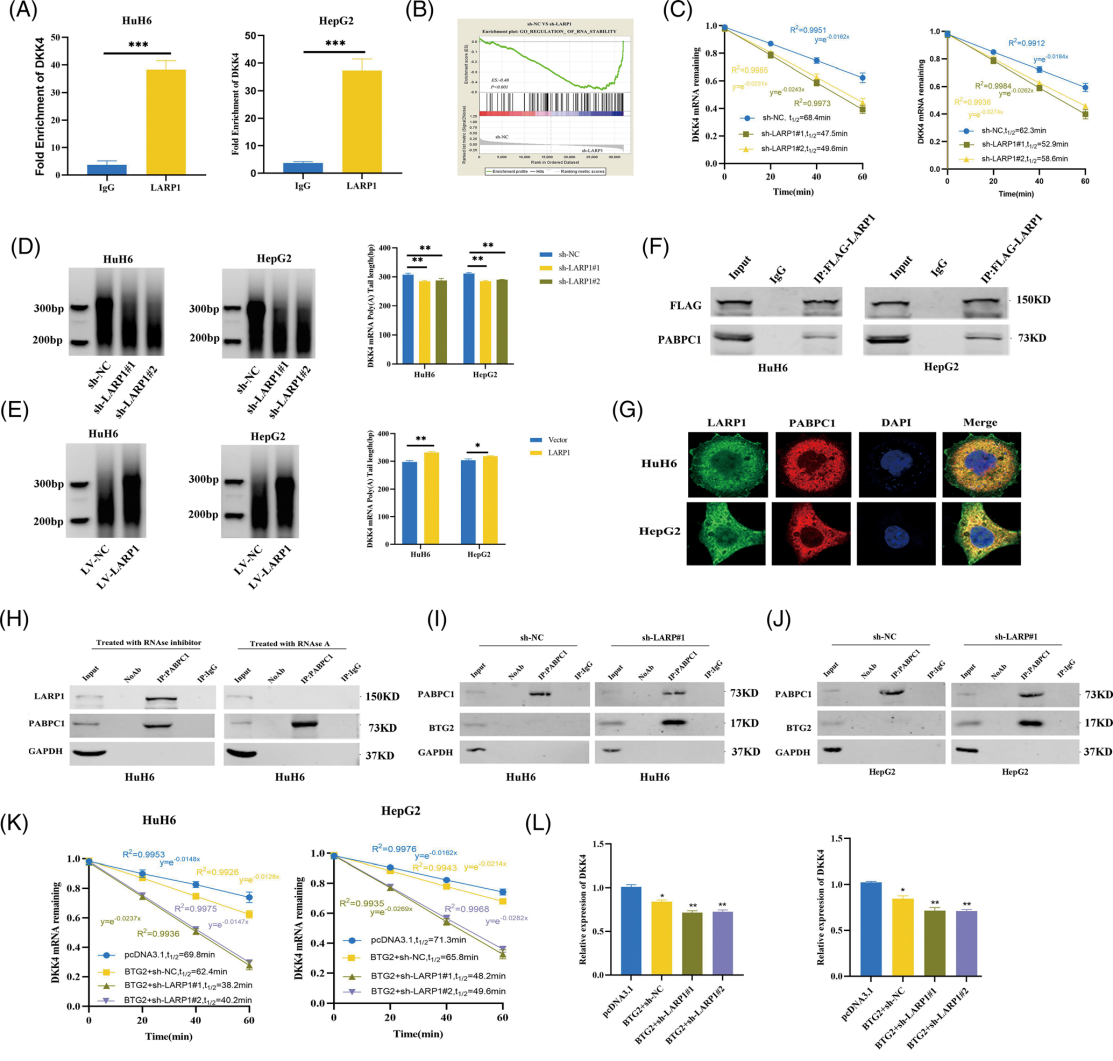
La‐related protein 1 (LARP1) stabilises DKK4 mRNA by competing with B‐cell translocation gene 2 (BTG2) for binding to PABPC1. (A) RNA immunoprecipitation (RIP)‐qPCR was conducted to detect DKK4 mRNA associated with LARP1 in hepatoblastoma (HB) cells, using IgG as a negative control. (B) GSEA analysis of gene sets related to RNA stability regulation in HuH6/sh‐NC and HuH6/sh‐LARP1#1 cells. (C) DKK4 mRNA level of HB/sh‐NC and HB/sh‐LARP1#1, #2 cells was detected by qRT‐PCR after treatment with actinomycin D for indicated times. (D and E) Rapid amplification of cDNA ends‐poly(A) test (RACE‐PAT) analyses for DKK4 mRNA poly(A)‐tail length in HB/sh‐NC and HB/sh‐LARP1#1, #2 cells (D) or HB/LV‐NC and HB/LV‐LARP1 cells (E). (F) Proteins from HB cells transfected with Flag‐LARP1 were immunoprecipitated using Flag antibody, followed by Western blotting using Flag and PABPC1 antibodies. (G) IF assays confirmed the co‐localisation of LARP1 and PABPC1 in HB cells. (H) Proteins from HuH6 cells treated with RNase inhibitor or RNase A were immunoprecipitated using PABPC1 antibody, followed by Western blotting using LARP1 and PABPC1 antibodies. (I and J) Proteins from HuH6 (I) and HepG2 (J) cells infected with sh‐NC or sh‐LARP1#1 were immunoprecipitated using PABPC1 antibody, followed by Western blotting using PABPC1 and BTG2 antibodies. (K and L) DKK4 mRNA level of HB/sh‐NC and HB/sh‐LARP1#1, #2 cells transfected with pcDNA3.1 or pcDNA3.1‐BTG2 vector was detected by qRT‐PCR after treatment with actinomycin D for indicated times (K) or not (L). *p < .05, **p < .01 and ***p < .001 between indicated groups.

LARP1 acts as an RNA stability regulator by directly interacting with RNA.^16^, ^18^ Through RNA immunoprecipitation (RIP)‐qPCR assays, we observed that DKK4 mRNA was enriched with LARP1 antibody compared with the IgG group, suggesting that LARP1 can bind to DKK4 mRNA (Figure 4A). Considering that gene sets related to RNA stability regulation were decreased in HuH6 cells stably silencing LARP1 by gene set enrichment analysis (Figure 4B), we speculated that LARP1 may upregulate DKK4 mRNA levels. mRNA stability assays confirmed that LARP1 deficiency was associated with a shorter DKK4 mRNA half‐life (Figure 4C). Because the poly(A)‐tail length is critical for mRNA stability control,^23^ we measured the poly(A)‐tail length of DKK4 mRNA. The results showed that the poly(A)‐tail was significantly shortened when LARP1 was silenced but elongated when LARP1 was overexpressed (Figure 4D,E). LARP1 has been reported to stabilise mRNA by protecting the poly(A)‐tail length depending on its interaction with PABPC1.^24^ To clarify whether PABPC1 is involved in LARP1‐mediated DKK4 upregulation, we performed co‐immunoprecipitation (co‐IP) assays and found that LARP1 interacted with PABPC1; however, this interaction was impaired by RNase A treatment but maintained by an RNase inhibitor, suggesting that the LARP1‐PABPC1 interaction was RNA‐dependent (Figure 4F,H and Figure S5A). Consistently, IF assays confirmed the colocalisation of LARP1 and PABPC1 (Figure 4G).

PABPC1 can also associate with B‐cell translocation gene 2 (BTG2), a member of the BTG/transducer of ERBB2 (BTG/TOB) family that activates mRNA deadenylation and degradation.^25^ Using co‐IP assays, we found that LARP1 blocked PABPC1 binding to BTG2, but LARP1 knock‐down allowed this interaction (Figure 4I,J). BTG2 overexpression weakened DKK4 mRNA stability and reduced mRNA levels, and LARP1 deficiency in these cells further decreased its stability (Figure 4K,L). mRNA deadenylation is catalysed by deadenylases such as the CCR4‐NOT complex, PAN2‐PAN3 complex and PARN.^26^ To investigate which deadenylase was involved, we used specific siRNAs targeting these enzymes (Figure S4A–D). Only CNOT1 depletion elongated the poly(A)‐tail of DKK4 mRNA (Figure S4E,F). Co‐IP assays showed that PABPC1 interactions with CNOT1, CAF1 and CCR4A were not hindered with RNase inhibitor treatment but disrupted by RNase A treatment, indicating that PABPC1 is associated with the CCR4‐NOT complex in an RNA‐dependent manner (Figure S5B,C). Furthermore, BTG2 depletion blocked the binding of PABPC1 to CCR4‐NOT complex subunits, suggesting that PABPC1 required BTG2 to recruit the CCR4‐NOT complex to the mRNA poly(A)‐tail (Figure S5D,E). Consequently, LARP1 protects DKK4 mRNA from deadenylation and decay by competing with BTG2 for PABPC1 binding.

### 3.5 The Ser672 residue of LARP1 is *O*‐GlcNAc‐modified by OGT

**FIGURE 5:**
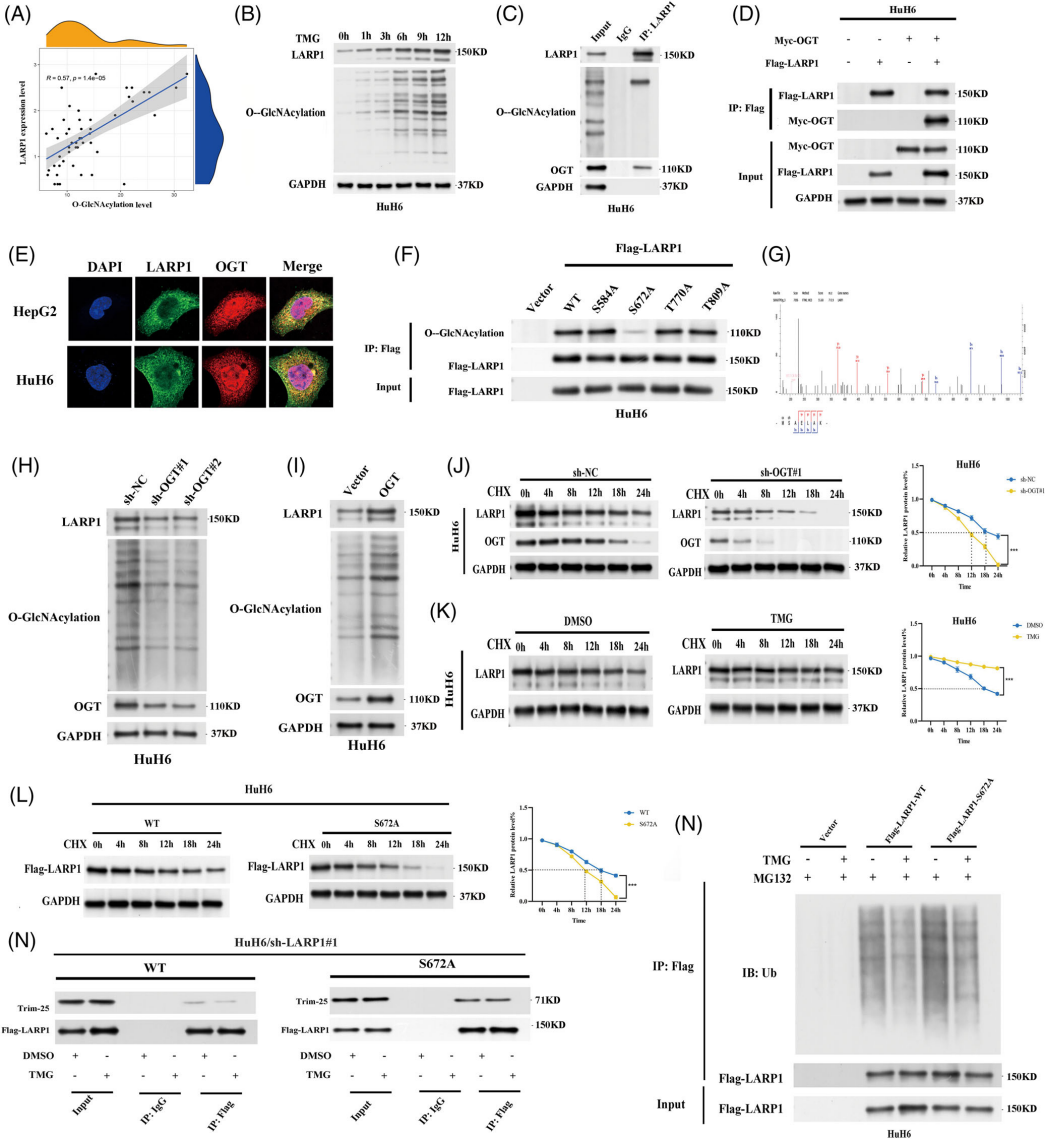
*O*‐GlcNAcylation of La‐related protein 1 (LARP1) Ser672 inhibits LARP1‐tripartite motif protein 25 (TRIM‐25) interaction to prevent ubiquitin/proteasome‐mediated LARP1 proteolysis. (A) Pearson correlation analysis of *O*‐GlcNAcylation and LARP1 protein level in 50 paired hepatoblastoma (HB) and normal tissues. (B) Western blotting analyses for *O*‐GlcNAcylation and LARP1 protein levels in HuH6 cells treated with TMG for indicated time points. (C) Proteins from HuH6 cells were immunoprecipitated using LARP1 antibody, followed by Western blotting using LARP1, *O*‐GlcNAc and *O*‐GlcNAc transferase (OGT) antibodies. (D) Proteins from HuH6 cells transfected with Myc‐OGT and/or Flag‐LARP1 were immunoprecipitated using Myc or Flag antibody, followed by Western blotting using Myc and Flag antibodies. (E) IF assays confirmed the co‐localisation of LARP1 and OGT in HB cells. (F) Proteins from HuH6 cells transfected with Flag‐LARP1‐wild‐type (WT), Flag‐LARP1‐S584A/S672A/T770A/T809A or empty vector were immunoprecipitated using Flag antibody, followed by Western blotting using *O*‐GlcNAc and Flag antibodies. (G) ETD mass spectra of peptides from LARP1. (H and I) Western blotting analyses for LARP1, *O*‐GlcNAcylation and OGT protein levels in HB/sh‐NC and HB/sh‐OGT#1, #2 cells (H) or HB/vector and HB/LV‐OGT cells (I). (J) LARP1 and OGT protein levels of HuH6 cells infected with sh‐NC or sh‐OGT#1 were detected by Western blotting assays after treatment with cycloheximide for indicated time points. (K and L) LARP1 protein level of HB cells treated with TMG or DMSO (K) or transfected with Flag‐LARP1‐WT or Flag‐LARP1‐S672A (L) was detected by Western blotting assays after treatment with cycloheximide for indicated time points. (M) Proteins from MG‐132 containing HuH6 cells transfected with Flag‐LARP1‐WT, Flag‐LARP1‐S672A or empty vector and treated with/without TMG were immunoprecipitated using Flag antibody, followed by Western blotting using Ubiquitin and Flag antibodies. (N) Proteins from HuH6/sh‐ALRP1# cells transfected with Flag‐LARP1‐WT or Flag‐LARP1‐S672A and treated with TMG or DMSO were immunoprecipitated using Flag antibody, followed by Western blotting using Flag and TRIM‐25 antibodies. ***p < .001 between indicated groups.

We conducted Pearson's correlation analysis and found that total *O*‐GlcNAcylation levels were positively correlated with LARP1 protein levels in 50 HB tissues (Figure 5A). By using an OGA inhibitor, both LARP1 and total *O*‐GlcNAcylation levels were significantly increased (Figure 5B). Next, we performed co‐IP assays and observed that endogenous LARP1 was *O*‐GlcNAc‐modified and interacted with OGT (Figure 5C and Figure S7A). HB cells transfected with Myc‐OGT and/or Flag‐LARP1 were subjected to co‐IP assays and identified that exogenous LARP1 and OGT interacted (Figure 5D and Figure S7B). IF assays further confirmed the colocalisation of LARP1 and OGT (Figure 5E).

To investigate which LARP1 domain binds to OGT, we constructed a panel of plasmids expressing the full‐length (Flag‐LARP1‐WT) or truncated LARP1 protein (Flag‐LARP1‐Del‐A/B/C/D). Co‐IP assays confirmed that LARP1 binds to OGT through its RRM‐L5 domain (Figure S7E). The *O*‐GlcNAcylated site of LARP1, detected by LC‐MS/MS, was Ser672 (Figure 5G). We also acquired three experimentally identified *O*‐GlcNAcylated sites (Ser584, Thr770 and Thr809) near the LARP1 RRM‐L5 domain from the *O*‐GlcNAcAtlas database (Figure S6A). To verify whether *O*‐GlcNAcylation occurs at these residues in HB, we generated a panel of plasmids encoding point mutants of the LARP1 protein (Flag‐LARP1‐S584A/S672A/T770A/T809A). HuH6 cells transfected with the individual mutant plasmids were subjected to co‐IP assays. Only the S672A mutant exhibited significantly lower *O*‐GlcNAcylation levels than WT LARP1, suggesting that Ser672 is the *O*‐GlcNAcylated site (Figure 5F). Collectively, these data confirmed that OGT interacts with the LARP1 RRM‐L5 domain to catalyse *O*‐GlcNAcylation of LARP1 Ser672.

### 3.6 *O*‐GlcNAcylation of LARP1 Ser672 protects LARP from degradation in a ubiquitin‐proteasome manner

Given that *O*‐GlcNAcylation is crucial for modulating protein abundance and function,^27^ we speculated that this modification might be involved in controlling LARP1 levels. Hence, we stably knocked down OGT in HB cells and observed reduced LARP1 (Figure 5H and Figure S7C). In contrast, OGT overexpression both led to increases in total *O*‐GlcNAcylation and LARP1 levels (Figure 5I and Figure S7D). To further elucidate whether *O*‐GlcNAcylation affects LARP1 upregulation, HB cells stably silenced for OGT were exposed to a protein synthesis inhibitor CHX. OGT knock‐down resulted in a shorter LARP1 protein half‐life (Figure 5J and Figure S7F). Intriguingly, LARP1 protein half‐life was significantly increased by TMG treatment (Figure 5K and Figure S7G) but was shortened in cells expressing the LARP1‐S672A mutant protein (Figure 5L and Figure S7H), suggesting that *O*‐GlcNAcylation of LARP1 at Ser672 enhanced its stability.

**FIGURE 6:**
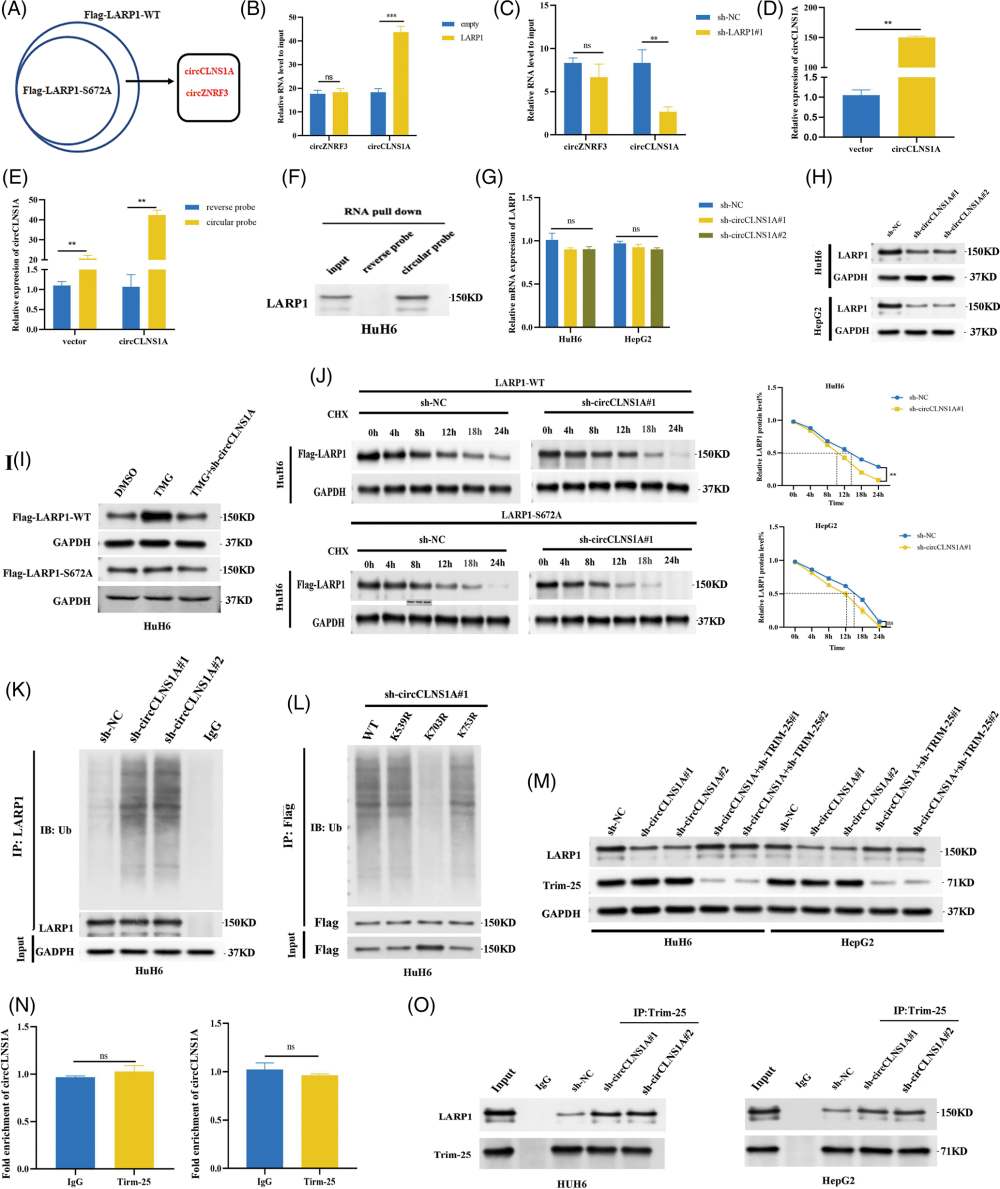
CircCLNS1A stabilises La‐related protein 1 (LARP1) protein by competing with tripartite motif protein 25 (TRIM‐25) for binding to LARP1. (A) RNA immunoprecipitation (RIP)‐seq was conducted in HuH6 cells transfected with Flag‐LARP1‐wild‐type (WT) or Flag‐LARP1‐S672A to identify RNAs associated with WT‐LARP1 or LARP1‐S672A mutant protein. (B and C) RIP‐qPCR was conducted to detect circCLNS1A and circZNRF3 associated with LARP1 in HuH6/vector and HuH6/LV‐LARP1 cells (B) or HuH6/sh‐NC and HuH6/sh‐LARP1#1 cells (C). (D) circCLNS1A level in HuH6/vector and HuH6/LV‐circCLNS1A was detected by qRT‐PCR. (E and F) RNA pull‐down with circCLNS1A probe or reverse probe was performed in HuH6 cells with indicated treatment, followed by qRT‐PCR (E) and Western blotting (F) to examine the enrichment of circCLNS1A and LARP. (G and H) LARP1 mRNA (G) and protein (H) levels in hepatoblastoma (HB)/sh‐NC and HB/sh‐circCLNS1A#1, #2 cells were detected by qRT‐PCR and Western blotting assays. (I) Western blotting analyses for WT‐LARP1 or LARP1‐S672A mutant protein levels in HuH6 cells treated with DMSO, TMG or TMG plus sh‐circCLNS1A#1. (J) Flag‐LARP1 protein level of HuH6/sh‐NC and HuH6/sh‐circCLNS1A#1 cells transfected with Flag‐LARP1‐WT or Flag‐LARP1‐S672A was detected by Western blotting assays after treatment with cycloheximide for indicated time points. (K) Proteins from HuH6/sh‐NC and HuH6/sh‐circCLNS1A #1, #2 cells were immunoprecipitated using LARP1 antibody, followed by Western blotting using Ubiquitin and LARP1 antibodies. (L) Proteins from HuH6/sh‐circCLNS1A#1 cells transfected with Flag‐LARP1‐WT or Flag‐LARP1‐K549R/K703R/K753R were immunoprecipitated using Flag antibody, followed by Western blotting using ubiquitin and Flag antibodies. (M) Western blotting analyses for LARP1 and TRIM‐25 protein levels in HB cells with indicated treatment. (N) RIP‐qPCR was conducted to detect circCLNS1A associated with TRIM‐25 in HB cells. (O) Proteins from HB/sh‐NC and HB/sh‐circCLNS1A#1, #2 cells were immunoprecipitated using LARP1 or TRIM‐25 antibody, followed by Western blotting using LARP1 and TRIM‐25 antibodies. **p < .01 and ***p < .001 between indicated groups.

The ubiquitin‐proteasome system is responsible for cellular protein degradation.^28^ To investigate whether *O*‐GlcNAcylation prevents LARP1 ubiquitination, cells transfected with Flag‐LARP1‐WT, Flag‐LARP1‐S672A or control vector were exposed to the proteasome inhibitor MG‐132 with or without TMG, and the results showed that TMG treatment decreased WT‐LARP1 ubiquitination level, whereas it had no effect on LARP1‐S672A, implying that LARP1 Ser672 *O*‐GlcNAcylation inhibited its ubiquitination (Figure 5M). To identify the ubiquitin ligase, we used Flag antibody and identified that tripartite motif protein 25 (TRIM‐25) had higher affinity to LARP1‐S672A than WT‐LARP1 by mass spectrometry analysis (Figure S6B). The E3 ubiquitin ligase TRIM‐25 catalyses the addition of polyubiquitin chains to its substrates for proteasomal degradation.^29^ Consistently, co‐IP assays confirmed that TRIM‐25 binding to LARP1‐S672A was stronger than WT‐LARP1. Moreover, TMG treatment impaired the interaction between TRIM‐25 and WT‐LARP1 but not between TRIM‐25 and LARP1‐S672A (Figure 5N). In conclusion, *O*‐GlcNAcylation of LARP1 Ser672 repressed the LARP1‐TRIM‐25 interaction and prevented ubiquitin/proteasome‐mediated proteolysis.

### 3.7 CircCLNS1A stabilises LARP1 protein by competing with TRIM‐25 for binding to LARP1

Several studies have shown that circRNAs play important roles in modulating LARP1 expression.^30^, ^31^ To screen the potential upstream effectors of LARP1, we performed RIP‐seq in HuH6 cells transfected with Flag‐LARP1‐WT or Flag‐LARP1‐S672A and identified two circRNAs, circCLNS1A and circZNRF3, which bind to WT‐LARP1 but not LARP1‐S672A (Figure 6A). RIP‐qPCR results demonstrated that only circCLNS1A interacted with LARP1 (Figure 6B,C). To investigate this finding, biotinylated circCLNS1A probes and reverse probes were used. The circCLNS1A probe pulled down more cirCLNS1A and LARP1 proteins compared to the control (Figure 6D–F). circCLNS1A deficiency significantly inhibited LARP1 protein levels but had no effect on LARP1 mRNA (Figure S7I,J and Figure 6G,H). Furthermore, circCLNS1A deficiency rescued increased WT‐LARP1 induced by TMG but had no influence on LARP1‐S672A (Figure 6I). To explore this finding, we performed protein stability assays. We found that circCLNS1A deficiency destabilised WT‐LARP1 and not LARP1‐S672A (Figure 6J). Moreover, circCLNS1A deficiency promoted LARP1 ubiquitination, suggesting that circCLNS1A functions in a ubiquitin‐proteasome‐related manner (Figure 6K). Considering that LARP1 dissociated from circCLNS1A after the Ser672 mutation, we hypothesised that circCLNS1A might react to LARP1 Ser672. We identified three ubiquitination sites (K549, K703 and K753) near Ser672 from the PhosphoSitePlus database (Figure S6C). To determine the ubiquitinated site, we generated plasmids encoding LARP1 point mutants and then transfected them into sh‐circCLNS1A#1 cells. Only the K703R mutant exhibited lower ubiquitination levels than WT‐LARP1, suggesting that K703 is the ubiquitination site (Figure 6L). Interestingly, we observed that TRIM‐25 depletion increased LARP1 protein levels by circCLNS1A knock‐down, implying that circCLNS1A regulates LARP1 protein via TRIM‐25 (Figure 6M). However, RIP‐qPCR revealed no interaction between TRIM‐25 and circCLNS1A (Figure 6N). To identify whether circCLNS1A competes with TRIM‐25 for LARP1 binding, we conducted co‐IP assays and found that circCLNS1A depletion enhanced the association between these proteins (Figure 6O).

### 3.8 Mutating LARP1 Ser672 inhibits its oncogenic effects on HB cells

**FIGURE 7:**
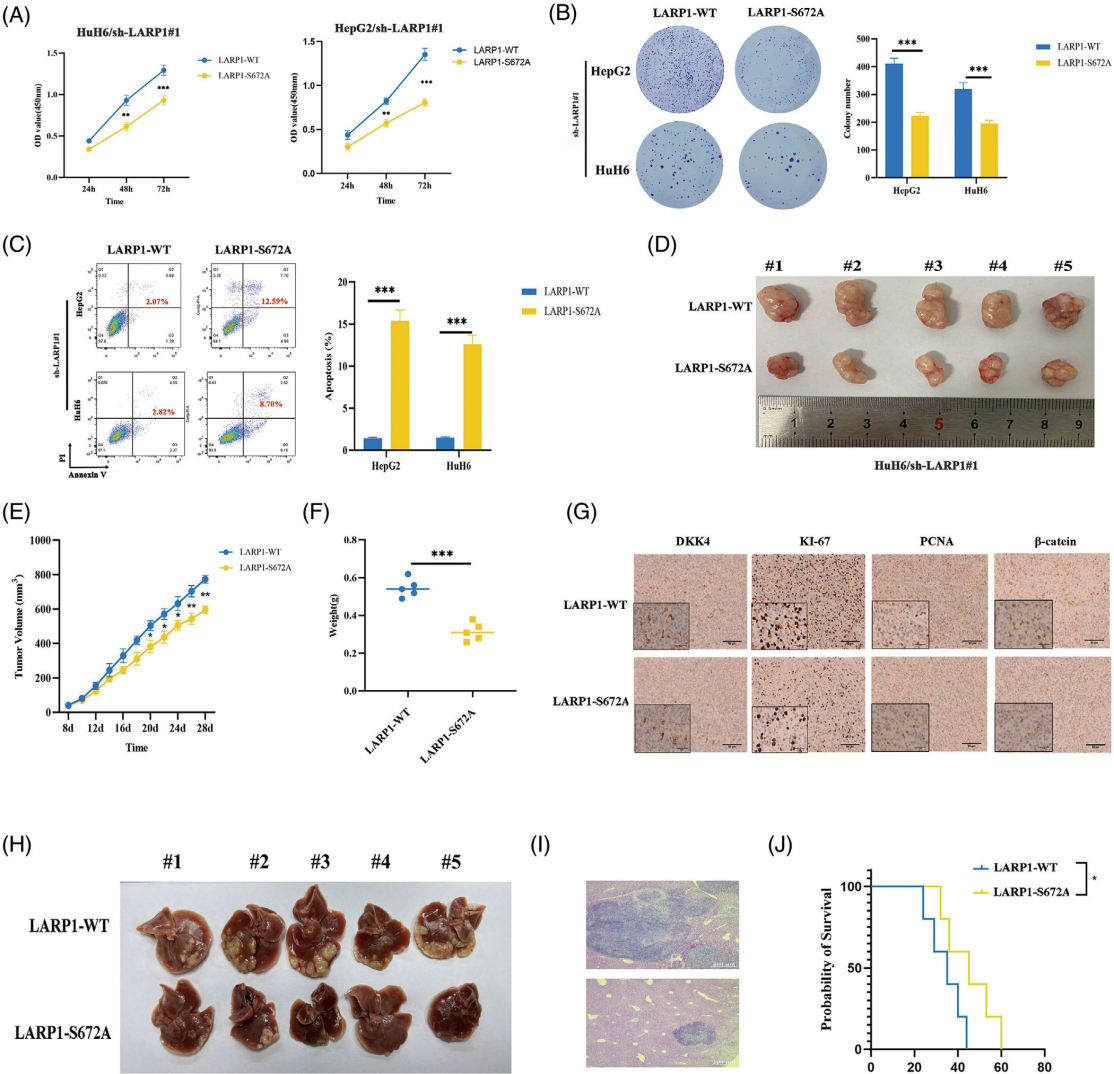
Mutating La‐related protein 1 (LARP1) Ser672 inhibits its oncogenic effects on hepatoblastoma (HB) cells. (A and B) The proliferative activities of HB/LV‐LARP1‐wild‐type (WT) or HB/LV‐LARP1‐S672A cells were examined using CCK‐8 (A) and colony formation (B) experiments. (C) Cell apoptosis analyses of HB/LV‐LARP1‐WT or HB/LV‐LARP1‐S672A cells by flow cytometry assays. (D) The stripped tumours from five nude mice subcutaneously implanted with HuH6/LV‐LARP1‐WT or HuH6/LV‐LARP1‐S672A cells. (E) Tumour growth curve was depicted by calculating tumour volumes on indicated days. (F) Average weight of stripped tumours. (G) Representative IHC images of DKK4, Ki‐67, PCNA and β‐catenin staining in stripped tumours. (H and I) The gross (H) and HE staining images (I) of nude mouse hepatoblastoma models in which HuH6/LV‐LARP1‐WT or HuH6/LV‐LARP1‐S672A cells were orthotopically injected into the liver. (J) Kaplan–Meier animal survival curves of the HB orthotopic mouse model. **p < .01 and ***p < .001 between indicated groups.

To uncover the biological role of *O*‐GlcNAcylated LARP1‐Ser672 in HB, we stably overexpressed LARP1‐WT or LARP1‐S672A mutant protein in HB/sh‐LARP1#1 cells. Both CCK‐8 and colony formation assays revealed that cell proliferation was significantly decreased in HB cells overexpressing LARP1‐S672A compared with LARP1‐WT (Figure 7A,B). Flow cytometry analysis showed that LARP1‐S672A promoted apoptosis (Figure 7C). To clarify Ser672's influence on tumour growth, HuH6/LARP1‐WT and HuH6/LARP1‐S672A cells were subcutaneously injected into the left flank and right flank of each nude mouse, respectively. The volume and weight of LARP1‐S672A tumours were markedly lower than those in the LARP1‐WT group (Figure 7D–F). Moreover, IHC assays showed that the staining intensities of DKK4, β‐catenin, Ki‐67 and PCNA were significantly weaker in the LARP1‐S672A group than in LARP1‐WT (Figure 7G). The results of an orthotopic mouse model showed that the livers of HuH6/LARP1‐WT mice had more tumour foci than those of the HuH6/LARP1‐MUT group (median survival, 34 days and 42 days, respectively; *p* = .041) (Figure 7H–J). Collectively, these results imply that the Ser672 mutation can inhibit LARP1's oncogenic role in HB.

### 3.9 Plasma DKK4 is a promising diagnostic and prognostic biomarker for HB

**FIGURE 8:**
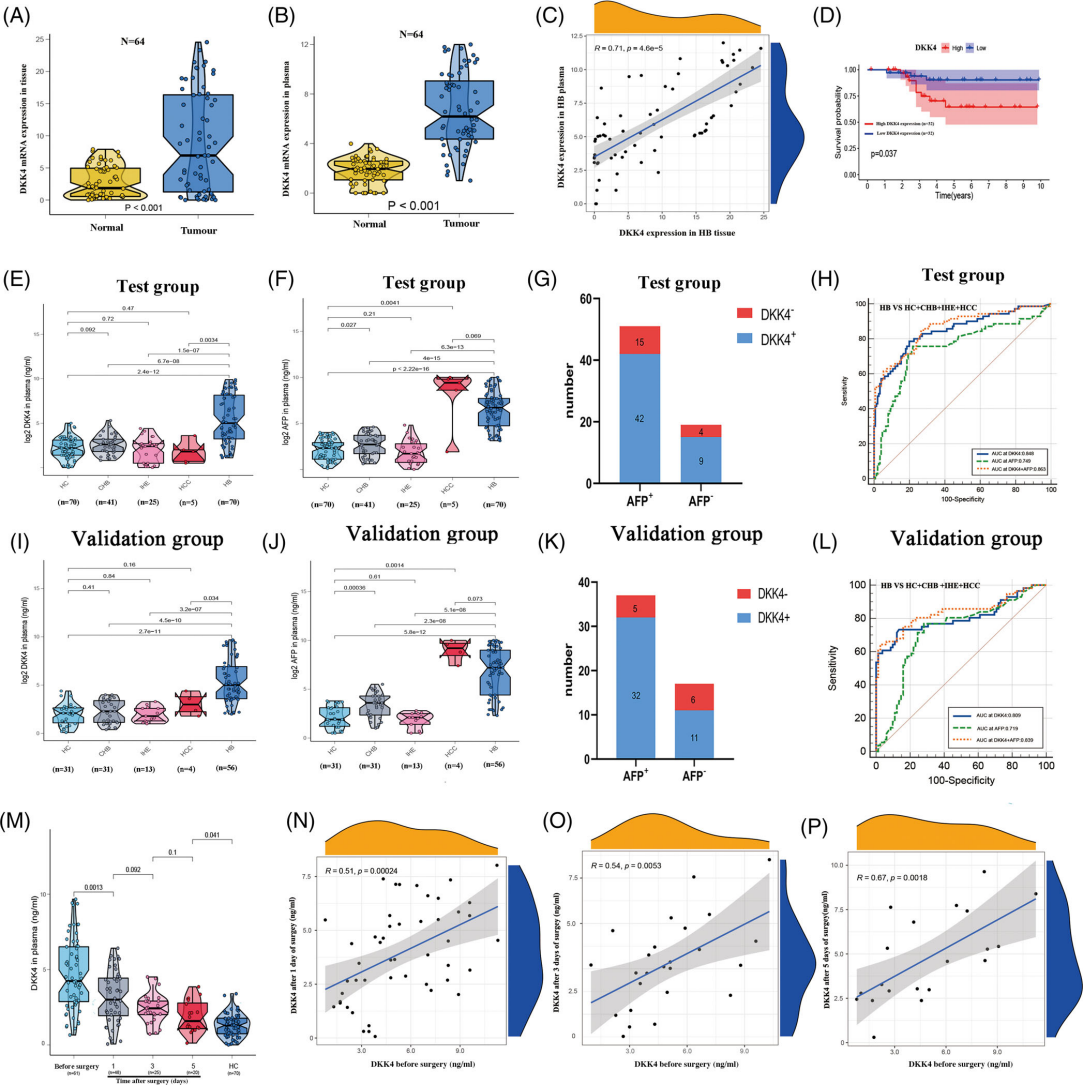
Plasma DKK4 is a promising diagnostic and prognostic biomarker for hepatoblastoma (HB). (A and B) qRT‐PCR assay for detecting the DKK4 mRNA expression in HB tissue (A) and plasma (B). (C) Pearson correlation analysis of DKK4 expression level in HB tissues and plasma. (D) Overall survival (OS) analysis of HB patients with high or low DKK4 expression. (E, F, I, J) ELISA assays for detecting plasma DKK4 and AFP levels of healthy candidates (HC) (*n* = 70), patients with CHB (*n* = 41), infantile hemangioendothelioma (IHE) (*n* = 25), hepatocellular carcinoma (HCC) (*n* = 14) and HB (*n* = 70) in the test cohort (E and F) or HC (*n* = 31), patients with CHB (*n* = 31), IHE (*n* = 13), HCC (*n* = 8) and HB (*n* = 56) in the validation cohort (I and J). (G and K) The number of DKK+ and DKK‐ patients in AFP+ and AFP‐ groups in the test (G) and validation (K) cohorts. (H and L) ROC curves for the diagnostic significance of plasma DKK4, plasma AFP or combination of plasma DKK4 and AFP for distinguishing HB from HC/CHB/IHE/HCC in the test (H) and validation (L) cohorts. (M) ELISA assays for detecting plasma DKK4 level in HC, HB patients before surgery and 1/3/5 day(s) post‐surgery. (N–P) Pearson correlation analyses of plasma DKK4 level in HB patients before surgery and 1 day post‐surgery (N), or 3 days post‐surgery (O) or 5 days post‐surgery (P).

To investigate the clinical value of DKK4 in HB, we conducted a multicentre study on DKK4 protein levels in HB samples. The results showed that DKK4 was significantly increased in patients with HB (Figure 8A,B). Pearson's correlation analysis analysed the consistency of DKK4 expression in HB tissue and serum (Figure 8C). Kaplan–Meier survival curve analysis confirmed that patients with high DKK4 expression in HB tissue had shorter OS than those with low expression (Figure 8D). To assess the diagnostic significance of plasma DKK4 in HB, we enrolled and randomly separated HC and patients with CHB, IHE, HCC or HB into the test or validation cohort. As shown in Figure 8E,I, in the two cohorts, plasma DKK4 levels were significantly higher in HB patients than in HC and CHB/IHE/HCC patients, but there was no obvious change in CHB/IHE/HCC patients compared to HC. Additionally, plasma AFP levels were elevated in HB patients compared to HC and CHB/IHE patients. As expected, HCC patients exhibited significantly higher AFP levels than HB patients in the two cohorts (Figure 8F,J). Subsequently, we categorised HB patients into two groups, AFP+ and AFP‐, and analysed the number of DKK+ and DKK‐ patients in the two groups. The number of DKK+ patients was higher than that of DKK‐ patients in both AFP groups (Figure 8G,K). To further evaluate the potential of DKK4 to only identify HB, we conducted ROC curve analysis and found that DKK4 showed a stronger diagnostic value (AUC_test_ = .848; AUC_validation_ = .809) than AFP (AUC_test_ = .749; AUC_validation_ = .719), whereas the combination of DKK4 and AFP exhibited the strongest diagnostic value (AUC_test_ = .863; AUC_validation_ = .839) (Figure 8H,L). Subsequently, we detected plasma DKK4 levels in patients before surgery and 1/3/5 day(s) post‐surgery and found that plasma DKK4 levels were lower in the post‐surgery groups versus the pre‐surgery group (Figure 8M). Moreover, the plasma DKK4 levels before surgery was correlated with those at 1/3/5 day(s) post‐surgery (Figure 8N–P). These findings indicate that plasma DKK4 may be a potential biomarker for HB diagnosis and prognosis.

## 4 DISCUSSION

In this study, we demonstrated that LARP1 is highly expressed in HB and is associated with poor prognosis. Proteomics and co‐IP analyses were performed to reveal *O*‐GlcNAcylation as a PTM of LARP1 that interacts with OGT in HB. Further studies showed that *O*‐GlcNAcylation hinders TRIM‐25 catalysed ubiquitination of LARP1 (Figure S8). In addition, we revealed that *O*‐GlcNAcylation at S672 of LARP1 plays an important tumour‐promoting role in HB by preventing its degradation.

Numerous recent studies on LARP1 have demonstrated that the La‐Module and the DM15 motif enable stability regulation of oncogenic TOP mRNAs.^15^, ^16^, ^24^ However, few studies have focused on LARP1 stabilizing or destabilizing other cancer‐related transcripts.^18^, ^32^ To identify LARP1's oncogenic mechanisms in HB biology, we conducted RNA‐seq in LARP1‐silenced cells and observed significant transcriptome alterations related to the Wnt signalling pathway, in which DKK4 ranked first. An overactive Wnt/β‐catenin signalling pathway is a hallmark of HB.^33^ This is mainly attributed to the recurrent CTNNB1 mutation found in 50%–90% of HB patients. It prevents β‐catenin degradation, leading to β‐catenin translocation to the nucleus where it activates downstream oncogenes with the transcription factor TCF/LEF.^34^ Beyond the CTNNB1 mutation, the Dickkopf (DKK) family is known for fine‐tuning Wnt activity.^35^ For example, DKK1 is defined as an agonist of the Wnt/β‐catenin signalling pathway in HCC^36^ but an antagonist in rhabdomyosarcoma^37^ and ovarian cancer.^38^ Similarly, DKK4 affects tumourigenesis and development by modulating the Wnt/β‐catenin signalling pathway. DKK4 inhibits HCC cell growth and invasion by both promoting β‐catenin degradation and inhibiting the expression of its downstream effectors CD44, cyclin D1 and c‐Jun.^39^ Here, we demonstrated that LARP1's oncogenic effects in HB cells were through increased DKK4 expression, which in turn promoted β‐catenin expression and nuclear import.

Poly(A)‐tail length control is a crucial means of regulating mRNA turnover through exoribonuclease‐catalysed degradation.^23^ In this study, we confirmed that LARP1 functions as a chaperone of DKK4 mRNA and protects its poly(A)‐tail length and stability. PABPC1, a cytoplasmic poly(A)‐tail‐bound protein, can be a positive or negative regulator of mRNA deadenylation.^40^, ^41^, ^42^ Here, we found that LARP1 interacts with PABPC1 in an RNA‐dependent manner. Other investigators have found a connection among BTG2, PABPC1 and the CCR4‐NOT deadenylase complex. For instance, BTG2 can simultaneously interact with PABPC1 and CAF1, which in turn stimulates CAF1‐mediated mRNA deadenylation.^43^ Coincidentally, our findings identified that the LARP1–PABPC1 interaction competitively inhibited PABPC1 binding to BTG2, which consequently disrupted the recruitment of the CCR4‐NOT complex to DKK4 mRNA, ultimately protecting DKK4 mRNA from deadenylation and degradation.

*O*‐GlcNAcylation and ubiquitination are two essential PTMs that control protein abundance and function.^27^, ^28^ Many studies have uncovered crosstalk between the two PTMs. For example, *O*‐GlcNAcylation of PGC‐1α recruits the deubiquitinating enzyme BAP1, thereby decreasing its ubiquitination and degradation.^44^ DOT1L *O*‐GlcNAcylation blocks its interaction with UBE3C and prevents ubiquitination.^45^ Here, we demonstrated that *O*‐GlcNAcylation of LARP1 Ser672 hampers the interaction between LARP1 and the E3 ubiquitin ligase TRIM‐25, thus preventing LARP1 ubiquitination and proteolysis. Although TRIM‐25 utilises circRNAs as a scaffold for efficient ubiquitination and substrate degradation,^46^ we found that it did not interact with circCLNS1A, which binds and upregulates LARP1. In fact, circCLNS1A competed with TRIM‐25 for LARP1 binding, thus blocking K703 ubiquitination. Furthermore, an Ser672 point mutation prevented circCLNS1A from binding to LARP1 and blocked its oncogenic effects in HB cells, which may be a therapeutic strategy.

The rarity, undetected onset and lack of effective serum biomarkers lead to delayed HB diagnosis, which in turn leads to poor prognosis.^1^, ^5^, ^6^ Serum AFP remains the standard for HB diagnosis, but its nonspecificity limits AFP as an ideal biomarker for HB.^7^, ^8^, ^9^ DKK4 is a secreted glycoprotein that has potential as a serum biomarker for cancer detection.^47^ Here, we confirmed that plasma DKK4 levels were higher in HB patients than in HC and patients with CHB/IHE/HCC but not between patients with CHB/IHE/HCC patients and HC. ROC curve analysis verified that plasma DKK4 had a stronger diagnostic value than plasma AFP for distinguishing only HB, whereas the combination of plasma DKK4 and AFP provided enhanced diagnostic accuracy. Additionally, Kaplan–Meier survival curve analysis confirmed that patients with high DKK4 expression had shorter OS than those with low expression. Therefore, DKK4 levels are a promising serum biomarker for HB diagnosis.

In conclusion, these findings provide a novel oncogenic mechanism of the circCLNS1A/LARP1/DKK4 cascade, a basis for targeting *O*‐GlcNAcylated LARP1 as a therapeutic strategy, and the potential of serum DKK4 as a diagnostic and prognostic biomarker for HB.

## CONFLICT OF INTEREST STATEMENT

The authors declare no conflicts of interest.

## Supporting information

Supporting Information

Supporting Information

Supporting Information

Supporting Information

Supporting Information

Supporting Information

Supporting Information

Supporting Information

Supporting Information

Supporting Information

Supporting Information
