# Supplementary material for: O‐GlcNAcylated LARP1 positively regulated by circCLNS1A facilitates hepatoblastoma progression through DKK4/β‐catenin signalling
Source: Clin Transl Med. 2023 Apr 17;13(4):e1239. doi: 10.1002/ctm2.1239 (PMC10111636; doi:10.1002/ctm2.1239)
Supplement: Supplementary file 1 — Supporting Information [file CTM2-13-e1239-s010.docx]

**Supplemental Tables**

**Table S1. Characterization of clincopathological features of 64 patients with HB**

| Parameters | Group | case |
| --- | --- | --- |
| Age(year)  Gender  AFP at diagnosis  LDH at diagnosis  Tumor size  PEWEWXT  Histology  Metastasis | ＜3  ≥3  Male  Female  ＜12000ng/ml  ≥12000ng/ml  ＜245U/L  ≥245U/L  NA  ＜500mm3  ≥500mm3  NA  Ⅰ-Ⅱ  Ⅲ-Ⅳ  NA  Wholly epithelial  Mixed epithelial mesenchymal  NA  Yes  NO  NA | 26  38  46  18  16  48  20  33  11  26  30  8  20  37  7  16  31  17  8  54  2 |

**Table S2. Chemical reagents used in this study**

| Chemical reagents | Vendors | Cat# |
| --- | --- | --- |
| actinomycin D  cycloheximide  TMG  MG-132  AFP ELISA Kit  Dkk-4 ELISA Kit  Lipofectamine 2000  TRIzol  PrimeScript RT Reagent Kit  RIPA lysis buffer | MedChemExpress  MedChemExpress  MedChemExpress  Selleck  RayBiotech Life  RayBiotech Life  Invitrogen  Invitrogen  Takara Bio  Beyotime | HY-17559  HY-12320  HY-12588  S2619  IQH-AFP-1  IQH-DKK4-1  [11668027](https://www.thermofisher.cn/order/catalog/product/cn/zh/11668027)  15596018  RR037B  P0013J |

**Table S3. Information for the expression vectors used in this study**

| Plasmids | Sources | Vectors |
| --- | --- | --- |
| Flag-LARP1-WT  Flag-LARP1-S584A  Flag-LARP1-S672A  Flag-LARP1-T770A  Flag-LARP1-T809A  Flag-LARP1- Del-A  Flag-LARP1- Del-B  Flag-LARP1- Del-C  Flag-LARP1- Del-D  Flag-LARP1-K539R  Flag-LARP1-K703R  Flag-LARP1-K753R  Myc-OGT  sh-LARP1#1  sh-LARP1#2  sh-OGT#1  sh-OGT#2  sh-Trim-25#1  sh-Trim-25#2  sh-circCLNS1A#1  sh-circCLNS1A#2 | Invitrogen  Invitrogen  Invitrogen  Invitrogen  Invitrogen  Invitrogen  Invitrogen  Invitrogen  Invitrogen  Invitrogen  Invitrogen  Invitrogen  Invitrogen  GenePharma  GenePharma  GenePharma  GenePharma  GenePharma  GenePharma  GenePharma  GenePharma | pcDNA3.1-3xFlag-C  pcDNA3.1-3xFlag-C  pcDNA3.1-3xFlag-C  pcDNA3.1-3xFlag-C  pcDNA3.1-3xFlag-C  pcDNA3.1-3xFlag-C  pcDNA3.1-3xFlag-C  pcDNA3.1-3xFlag-C  pcDNA3.1-3xFlag-C  pcDNA3.1-3xFlag-C  pcDNA3.1-3xFlag-C  pcDNA3.1-3xFlag-C  pCMV-Myc-C  pLVS-shRNA1  pLVS-shRNA1  pLVS-shRNA1  pLVS-shRNA1  pLVS-shRNA1  pLVS-shRNA1  pLVS-shRNA1  pLVS-shRNA1 |

**Table S4. Targeting sequences for siRNAs**

|  | Target Sequence |
| --- | --- |
| si-PARN#1  si-PARN#1  PAN2-siRNA1  PAN2-siRNA2  PAN3-siRNA1  PAN3-siRNA2  CNOT1-siRNA1  CNOT1-siRNA2 | GACCUUCAGUCUCUGCAUUAA  GGCAUUCAUGUUGAGACUUUA  CCUGCCUUCUUGCGCUUCAUU  GAGCCUGUACGCUGUCUAAUU  GUCUCACAGAUUCCUAUUU  CGACUUACUUCUAUACAGA  UGCCUAUUUGGUGGUAUAAUU  GCCAAAUUGUCUCGAAUACUU |

**Table S5. Targeting sequences for shRNAs**

|  | 5’ | STEM | LOOP | STEM | 3’ |
| --- | --- | --- | --- | --- | --- |
| sh-OGT#1  sh-OGT#2  sh-circCLNS1A#1  sh-circCLNS1A#2  sh-Trim25#1  sh-Trim25#2  sh-LARP1#1  sh-LARP1#2 | Ccgg  aattcaaaaa  Ccgg  aattcaaaaa  Ccgg  aattcaaaaa  Ccgg  aattcaaaaa | GCCCTAAGTTTGAGTCCAAAT  GCAGGAAGCTCTGATGCATTA  GCCTAGTGATAAATCAGCGTT  GCGTTGGAGGCAATGTTCACT  GCAAATGTTCCCAGCACAATC  GGTGGAGCAGCTACAACAAGA  GCCGGCCGTGCTGGGAGAGCA  GGAGAGCAGGCGAGCAGAACA | CTCGAG  CTCGAG  CTCGAG  CTCGAG  CTCGAG  CTCGAG  CTCGAG  CTCGAG | ATTTGGACTCAAACTTAGGG  TAATGCATCAGAGCTTCCTGC  AACGCTGATTTATCACTAGGC  AGTGAACATTGCCTCCAACGC  GATTGTGCTGGGAACATTTGC  TCTTGTTGTAGCTGCTCCACC  TCTTGTTGTAGCTGCTCCACC  TGTTCTGCTCGCCTGCTCTCC | TTTTTg  TTTTTg  TTTTTg  TTTTTg |

|  | Primers | Sequences |
| --- | --- | --- |
| LARP1  DKK4  CNOT1  PAN3  PARN  PAN2  circCLNS1A  Trim-25 | Forward  Reverse  Forward  Reverse  Forward  Reverse  Forward  Reverse  Forward  Reverse  Forward  Reverse  Forward  Reverse  Forward  Reverse | CAAGACACAGTTCAAACCCA  GTTTCCGCTCATTAAGGCAG  GGTACACTCTGTGTTGAACG  TCCCTTCATGCATGTGTGACC  CATTCCACATCTTGAGGCC  CTTCTGCTGTGGCGTATGTG  CAATCAATGAGAGGCCGGAG  GCACCTGCTTCTGTCACCTG  CTGCAGAATCACTATTACCGC  CAGGAATCGGTCTGCTCAAG  CCTTGCTTGGTACTTTCTGG  TTGTGGAAAGACTCAGGCTCA  GTGGAAGCACATGAAGAATC  GCTGATTTATCACTAGGCAC  CTGAGCTCCTGGAGTATTAC  ATCTCAGCCACAGAAGCTAC |

**Table S6. Primers for qPCR analyses**

**Table S7. Antibodies used in this study**

| Antibodies | Vendors | Cat# | Hosts | Working concentration |
| --- | --- | --- | --- | --- |
| LARP1  DKK4  β-catenin  FLAG  Myc  PABPC1  BTG2  O-GlcNAc  OGT  Ubiquitin  TRIM25  CNOT1  CAF1  CCR4A  IgG  GADPH  LAMINB  KI-67  PCNA  IRDye® 800CW Goat anti-Mouse IgG  IRDye® 800CW Goat anti-Rabbit IgG  Alexa Fluor® 488 AffiniPure Donkey Anti-Mouse IgG (H+L) Cy™3 AffiniPure Goat Anti-Rabbit IgG (H+L)Peroxidase AffiniPure Goat Anti-Mouse IgG (H+L)Peroxidase AffiniPure Goat Anti-Rabbit IgG (H+L) | Abcam  Proteintech  Abcam  CST  Cst  Proteintech  Santa Cruz  Abcam  Abcam  CST  Abcam  Proteintech  CST  Abcam  Millipore  Abcam  Abcam  CST  CST  LI-COR  LI-COR  Jackson  Jackson  Jackson  Jackson | Ab245635  27080-1-AP  ab16051  8146  2276  10970-1-AP  sc-517187  ab2739  ab96718  3936  ab86365  14276-1-AP  86665  ab54376  NI01  ab263962  ab16048  62548  2586  926-32210  926-32211  715-545-150  111-165-003  115-035-003  111-035-003 | Rabbit  Rabbit  Rabbit  Mouse  Mouse  Rabbit  Mouse  Mouse  Rabbit  Mouse  Rabbit  Rabbit  Rabbit  Mouse  Rabbit  Rabbit  Rabbit  Mouse  Mouse  Mouse  Rabbit  Mouse  Rabbit  Mouse  Rabbit | 1：1000  1：1000  1：1000  1：2000  1：2000  1：1000  1：100  1：1000  1：1000  1：1000  1：1000  1：1000  1：1000  1：1000  1：1000  1：2000  1：1000  1：100  1：100  1：2000  1：2000  1：500  1：500  1：500  1：500 |

**Table S8. The RACE-PAT sequence**

|  | Sequence |
| --- | --- |
| oligo (dT)-anchor | 5`-GCGAGCTCCGCGGCCGCG-T(12)-3` |
| DKK4 forward RACE | 5`-agcattgctcgattaatgag-3` |
| oligo (dT)-anchor PCR | 5`-GCGAGCTCCGCGGCCGCG-3` |
