# Supplementary figures and images for: O‐GlcNAcylated LARP1 positively regulated by circCLNS1A facilitates hepatoblastoma progression through DKK4/β‐catenin signalling
Source: Clin Transl Med. 2023 Apr 17;13(4):e1239. doi: 10.1002/ctm2.1239 (PMC10111636; doi:10.1002/ctm2.1239)

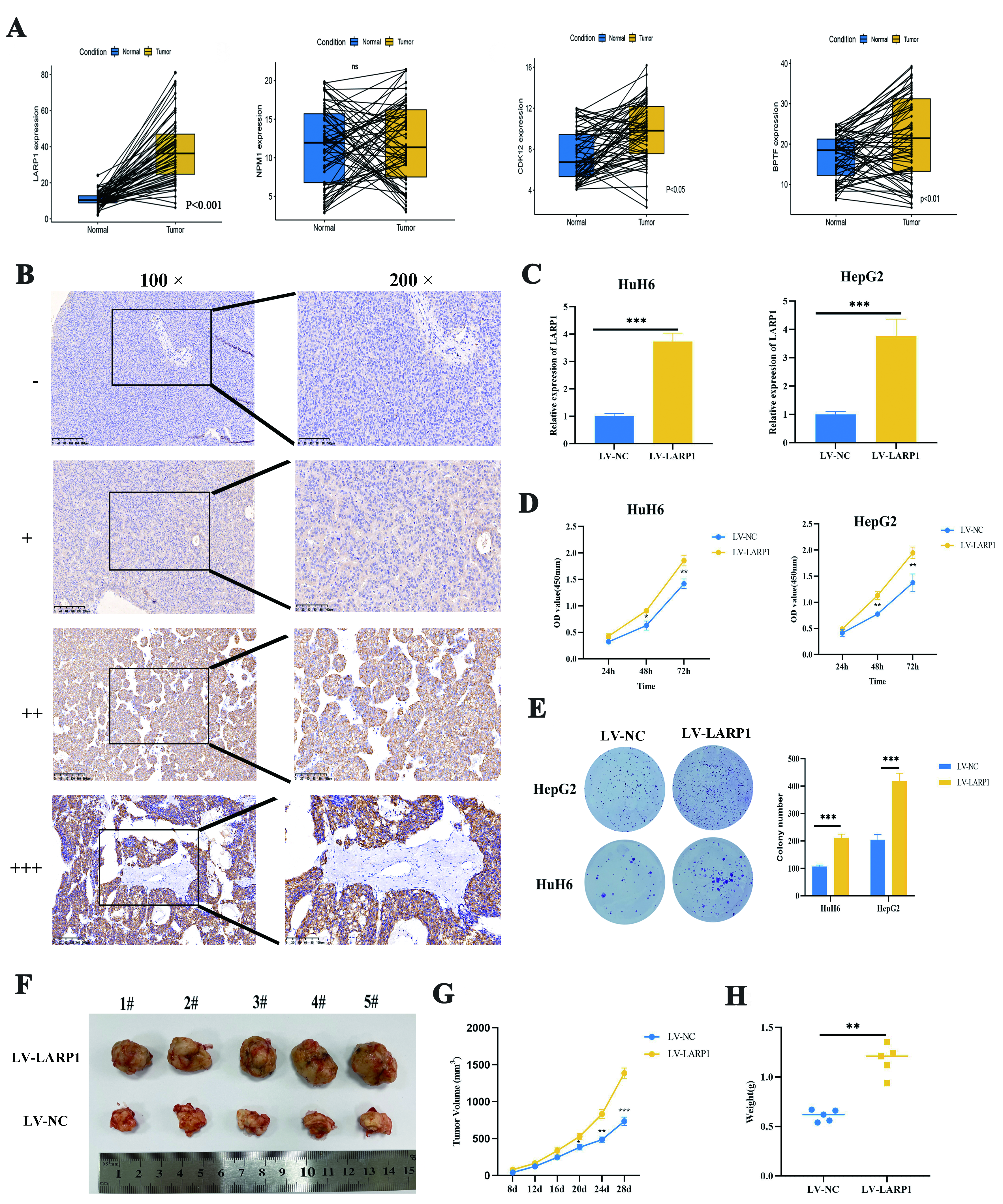

Supplement: Supplementary file 2 — Supporting Information [file CTM2-13-e1239-s006.tif]

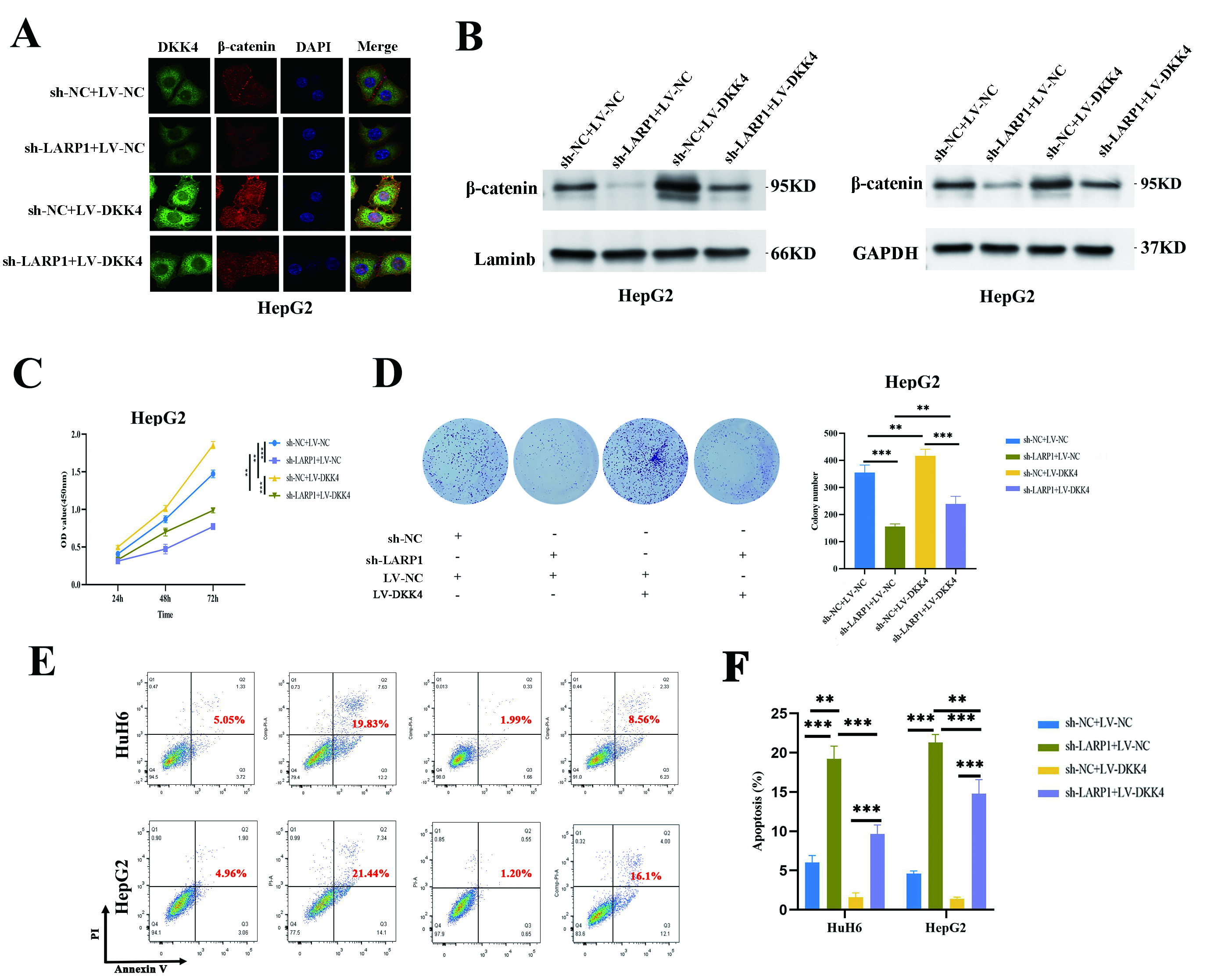

Supplement: Supplementary file 3 — Supporting Information [file CTM2-13-e1239-s008.tif]

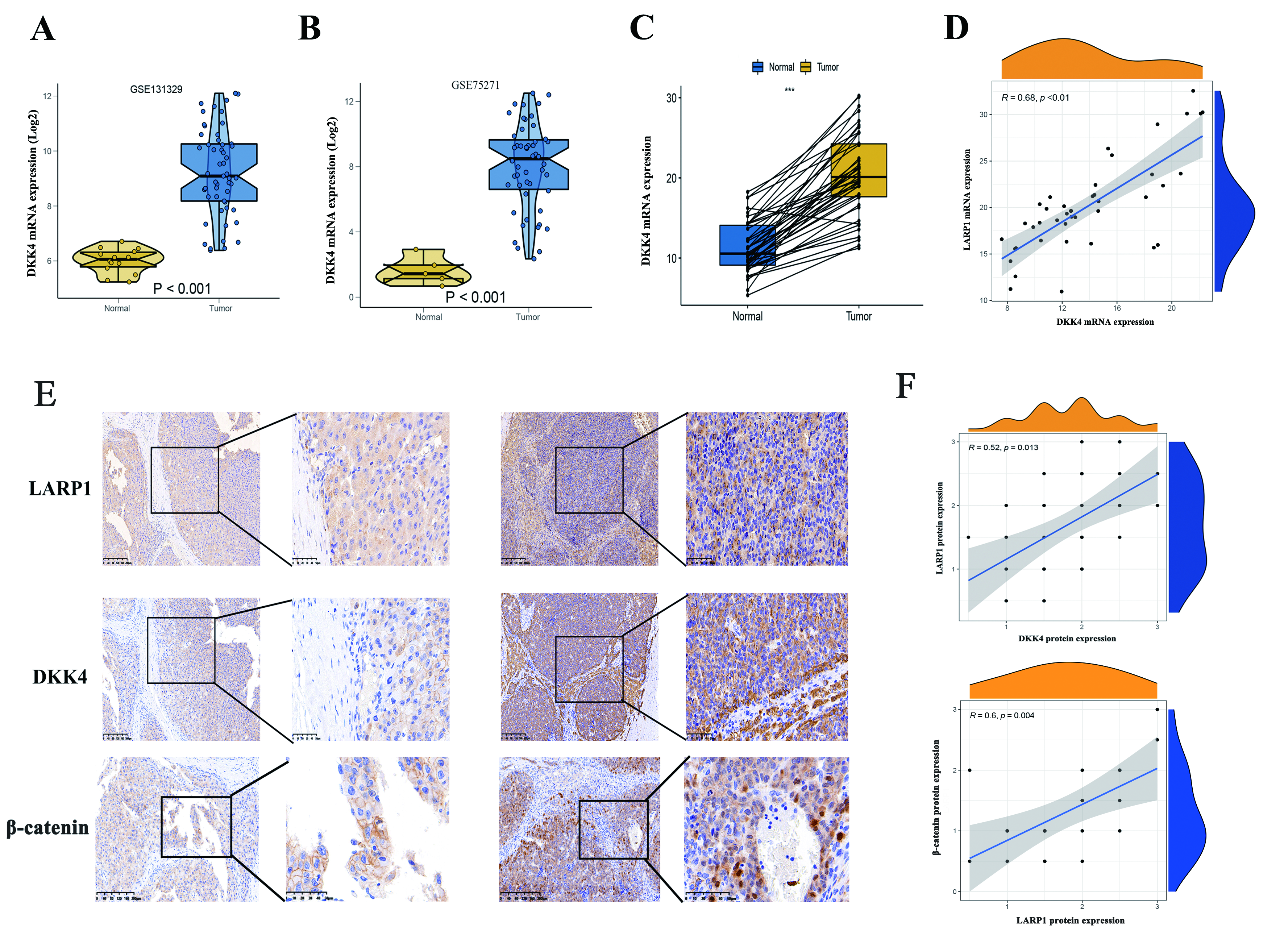

Supplement: Supplementary file 4 — Supporting Information [file CTM2-13-e1239-s004.tif]

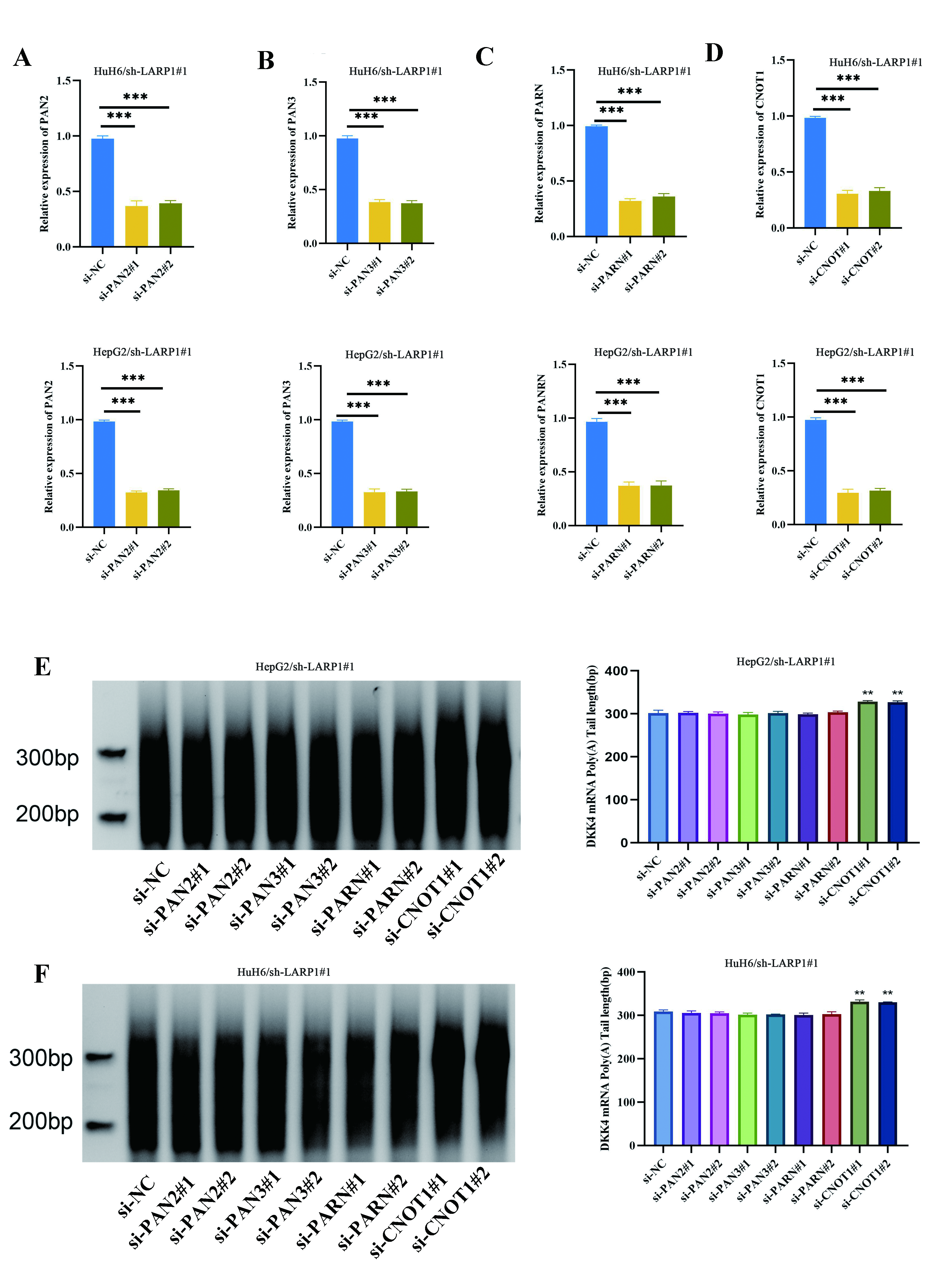

Supplement: Supplementary file 5 — Supporting Information [file CTM2-13-e1239-s003.tif]

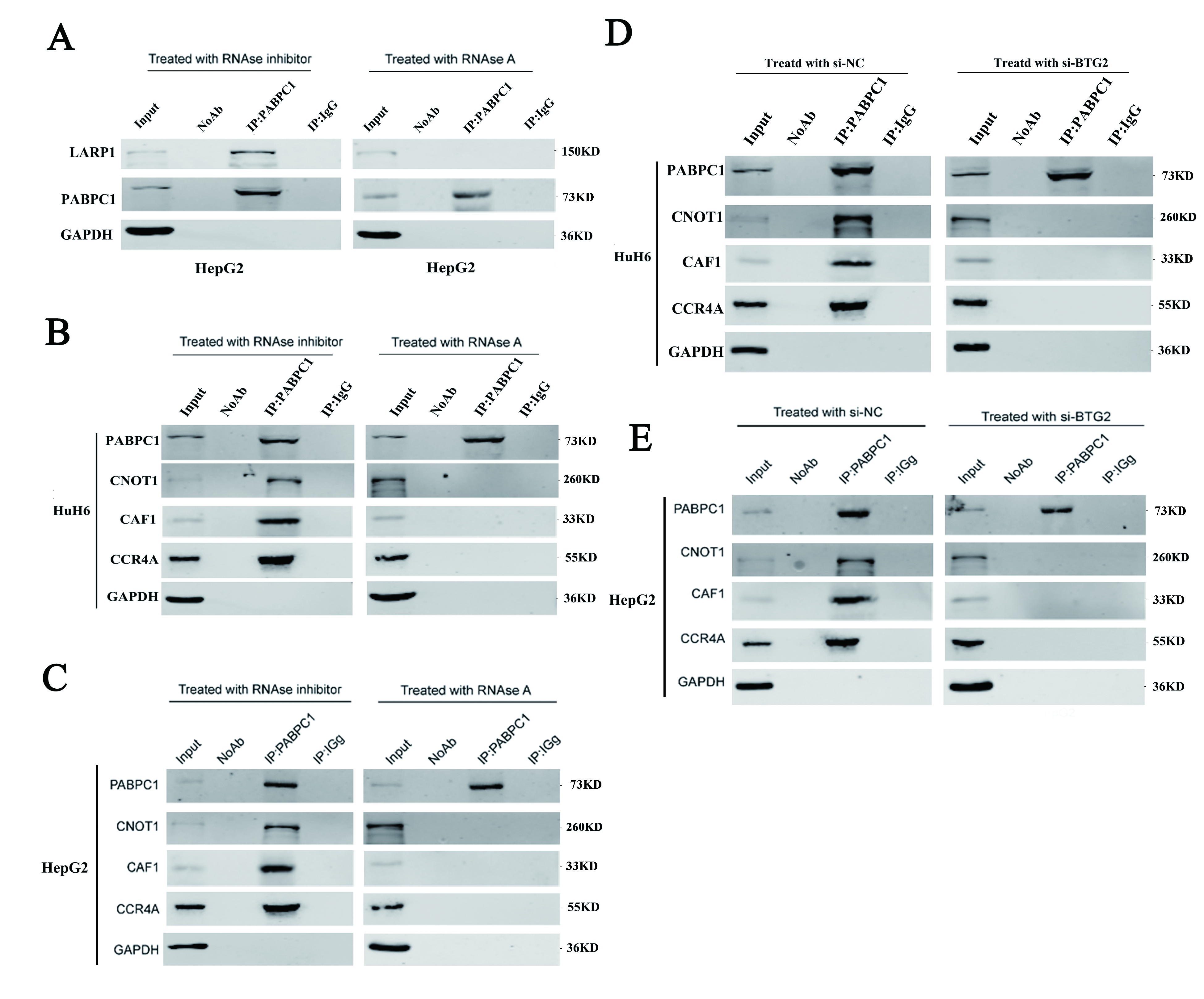

Supplement: Supplementary file 6 — Supporting Information [file CTM2-13-e1239-s011.tif]

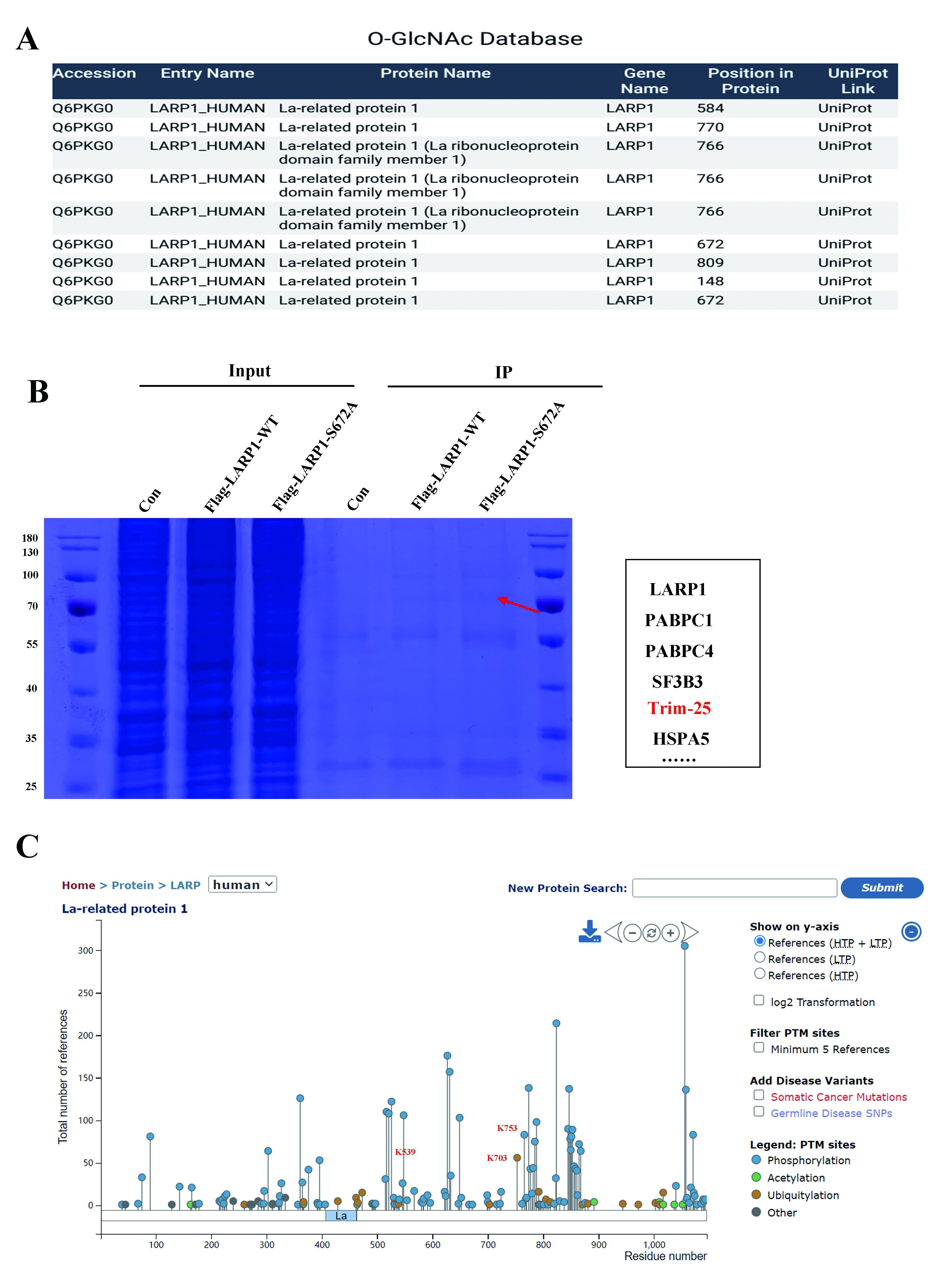

Supplement: Supplementary file 7 — Supporting Information [file CTM2-13-e1239-s009.tif]

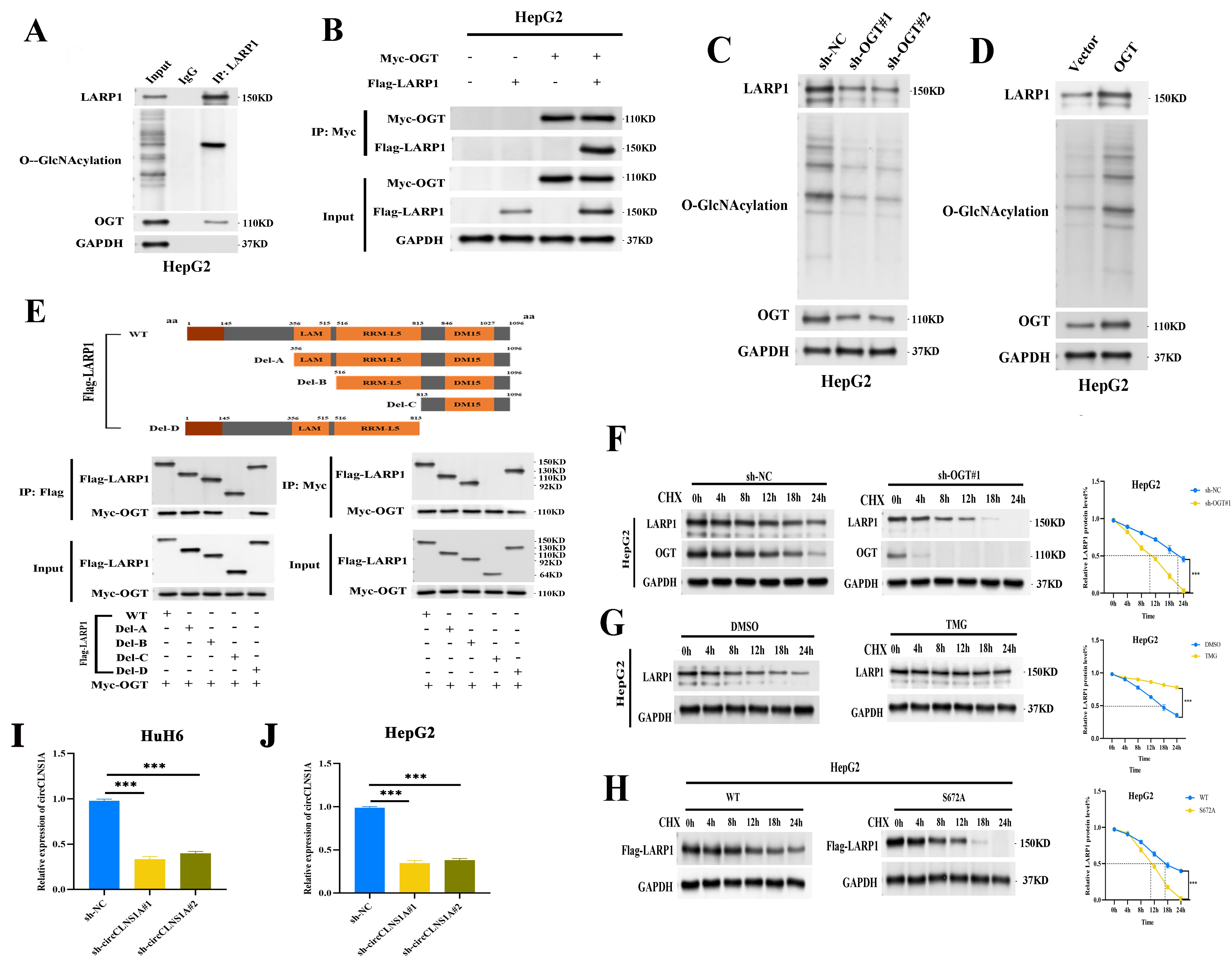

Supplement: Supplementary file 8 — Supporting Information [file CTM2-13-e1239-s002.tif]

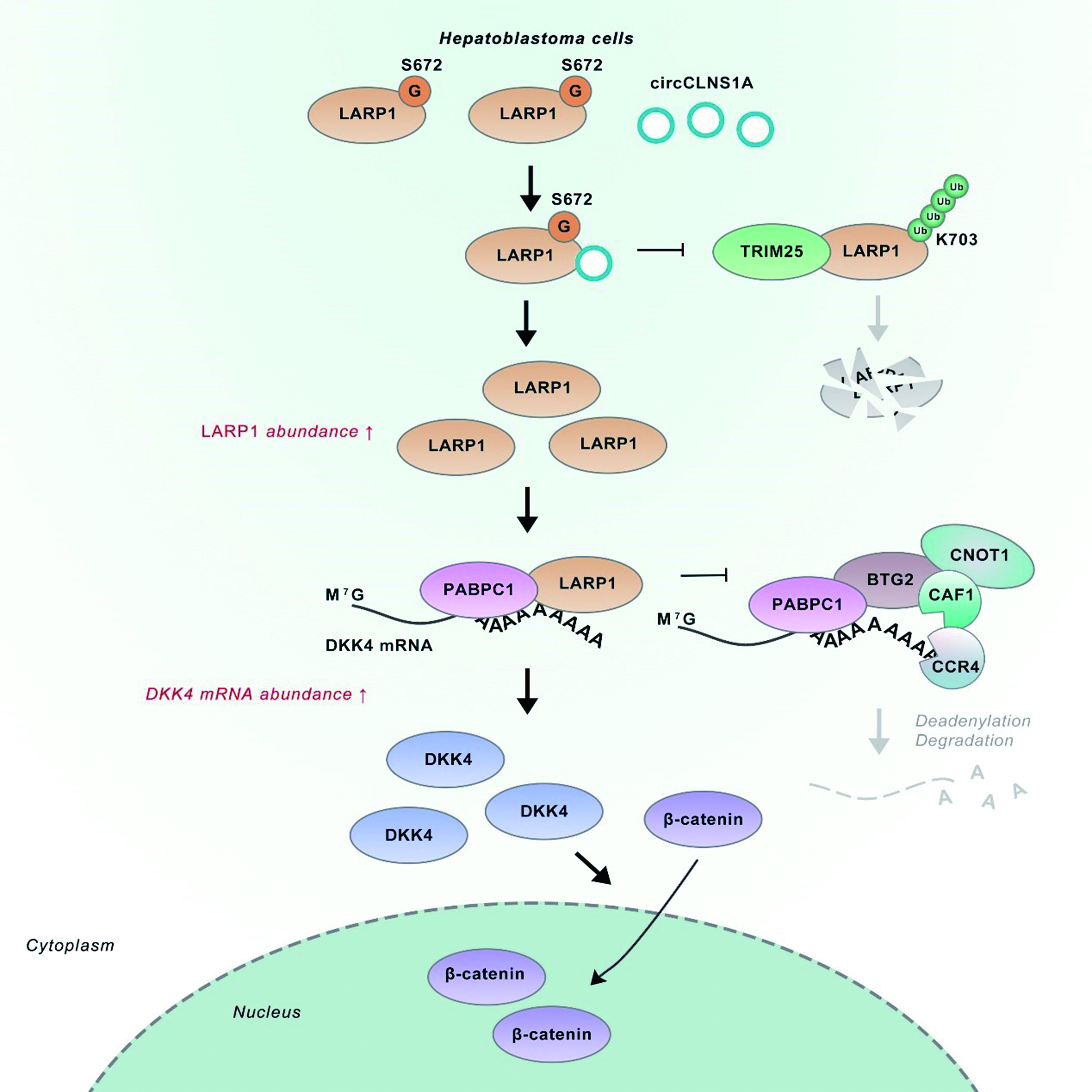

Supplement: Supplementary file 9 — Supporting Information [file CTM2-13-e1239-s001.tif]
