# Supplementary material for: O‐GlcNAcylated LARP1 positively regulated by circCLNS1A facilitates hepatoblastoma progression through DKK4/β‐catenin signalling
Source: Clin Transl Med. 2023 Apr 17;13(4):e1239. doi: 10.1002/ctm2.1239 (PMC10111636; doi:10.1002/ctm2.1239)
Supplement: Supplementary file 10 — Supporting Information [file CTM2-13-e1239-s005.docx]

**Supplementary figure legends**

**Supplementary Figure 1: LARP1 overexpression promotes HB cell proliferation and tumor growth.** (A) The LARP1, NPM1, CDK12 and BPTF mRNA expression in HB tissue was detected by qRT-PCR. (B) The criteria for definition of LARP1 protein level in HB tissues, determined by IHC assays, according to the percentage of cells exhibiting different staining intensities. (C) LARP1 mRNA level in HB/LV-NC and HB/LV-LARP1 cells was detected by qRT-PCR. (D-E) The proliferative activities of HB/LV-NC and HB/LV-LARP1 cells were examined using CCK-8 (D) and colony formation (E) experiments. (F) The stripped tumors from five nude mice subcutaneously implanted with HuH6/LV-NC and HuH6/LV-LARP1 cells. (G) Tumor growth curve was depicted by calculating tumor volumes on indicated days. (H) Average weight of stripped tumors. **P* < 0.05, ***P* < 0.01 and ****P* < 0.001 between indicated groups.

**Supplementary Figure 2: Oncogenic effects of LARP1 on HB cells are mediated by upregulation of DKK4 expression.** (A) HepG2/sh-NC and HepG2/sh-LARP1#1 cells co-infected with LV-NC or LV-DKK4 were adopted for IF assays to detect the expression and localization of DKK4 and β-catenin. (B) Western blotting analyses for β-catenin protein level in nuclear and cytoplasmic fractions of HepG2/sh-NC and HepG2/sh-LARP1#1 cells co-infected with LV-NC or LV-DKK4. (C-D) The proliferative activities of HepG2/sh-NC and HepG2/sh-LARP1#1 cells co-infected with LV-NC or LV-DKK4 were examined using CCK-8 (C) and colony formation (D) experiments. (E) Cell apoptosis analyses of HB/sh-NC and HB/sh-LARP1#1 cells co-infected with LV-NC or LV-DKK4 by flow cytometry assays. ***P* < 0.01 and ****P* < 0.001 between indicated groups.

**Supplementary Figure 3: DKK4 mRNA and protein levels are significantly increased in HB tissues.** (A-B) DKK4 mRNA level in normal (N) and HB (T) tissues from two GEO mRNA expression profiling datasets (GSE131329: N=14, T=53; GSE75271: N=5, T=50). (C) DKK4 mRNA level in our collection of 64 HB tissue specimens was detected by qRT-PCR. (D) Pearson correlation analysis of LARP1 and DKK4 mRNA levels in HB tissues. (E) Representative IHC images of LARP1, β-catenin and DKK4 staining in 21 paired HB and normal tissues (blue: DKK4 protein expression, brown: LARP1 protein expression). (F) Pearson correlation analysis of LARP1 and DKK4 or LARP1 and β-catenin protein levels in HB tissues. ****P* < 0.001 between indicated groups.

**Supplementary Figure 4:** **CNOT1 depletion elongates DKK4 mRNA poly(A)-tail.** (A-D) Indicated mRNA levels in HB/sh-LARP1#1 cells transfected with specific siRNAs against PAN2 (A), PAN3 (B), PARN (C) or CNOT1 (D) were detected by qRT-PCR. (E-F) RACE-PAT analyses for DKK4 mRNA poly(A)-tail length in HepG2 (E) and HuH6 (F) cells transfected with siRNAs against CNOT1, PAN2, PAN3 or PARN. ***P* < 0.01 and ****P* < 0.001 between indicated groups.

**Supplementary Figure 5: PABPC1 requires BTG2 to recruit CCR4-NOT complex to mRNA poly(A)-tail.** (A) Proteins from HepG2 cells treated with RNase inhibitor or RNase A were immunoprecipitated using PABPC1 antibody, followed by Western blotting using LARP1 and PABPC1 antibodies. (B-C) Proteins from HuH6 (B) and HepG2 (C) cells treated with RNase inhibitor or RNase A were immunoprecipitated using PABPC1 antibody, followed by Western blotting using PABPC1, CNOT1, CAF1 and CCR4A antibodies. (D-E) Proteins from HuH6 (D) and HepG2 (E) cells transfected with siNC or siBTG2 were immunoprecipitated using PABPC1 antibody, followed by Western blotting using PABPC1, CNOT1, CAF1 and CCR4A antibodies.

**Supplementary Figure 6: The PTM sites of LARP1.** (A) The experimentally identified O-GlcNAcylated sites of LARP1 obtained from O-GlcNAcAtlas Database. (B) Proteins from HuH6 cells transfected with Flag-LARP1-WT or Flag-LARP1-S672A were immunoprecipitated using Flag antibody, followed by Western blotting and mass spectrometry analysis. (C) The experimentally determined ubiquitination sites of LARP1 obtained from PhosphoSitePlus Database.

**Supplementary Figure 7: OGT interacts with LARP1 RRM-L5 domain to promote protein stability.** (A) Proteins from HepG2 cells were immunoprecipitated using LARP1 antibody, followed by Western blotting using LARP1, O-GlcNAc and OGT antibodies. (B) Proteins from HepG2 cells transfected with Myc-OGT and/or Flag-LARP1 were immunoprecipitated using Myc or Flag antibody, followed by Western blotting using Myc and Flag antibodies. (C-D) Western blotting analyses for LARP1, O-GlcNAcylation and OGT protein levels in HB/sh-NC and HB/sh-OGT#1, #2 cells (C) or HB/vector and HB/LV-OGT cells (D). (E) Proteins from Myc-OGT containing HB cells transfected with Flag-LARP1-WT or Flag-LARP1-Del-A/B/C/D were immunoprecipitated using Flag or Myc antibody, followed by Western blotting using Myc and Flag antibodies. (F) LARP1 and OGT protein levels of HepG2 cells infected with sh-NC or sh-OGT#1 were detected by Western blotting assays after treatment with cycloheximide for indicated time points. (G-H) LARP1 protein level of HB cells treated with TMG or DMSO (G) or transfected with Flag-LARP1-WT or Flag-LARP1-S672A (H) was detected by Western blotting assays after treatment with cycloheximide for indicated time points. (I-J) circCLNS1A expression levels in HB/sh-NC and HB/sh-circCLNS1A#1, #2 cells were detected by qRT-PCR. ****P* < 0.001 between indicated groups.

**Supplementary Figure 8:** **The proposed working model**

LARP1 is O-GlcNAcylated at the conserved S672 by OGT. Binding of circCLNS1A to O-GlcNAcylated LARP1 inhibits the ubiquitination degradation of K703 by Trim-25 and enhances its protein stability. The binding of LARP1 to PABPC1 competitively inhibits the degradation of DKK4 mRNA by BTG2-CCR4-NOT1, thereby activating the Wnt-β-catenin signalling pathway and promoting the malignant progression of HB.
