# Supplementary material for: O‐GlcNAcylated LARP1 positively regulated by circCLNS1A facilitates hepatoblastoma progression through DKK4/β‐catenin signalling
Source: Clin Transl Med. 2023 Apr 17;13(4):e1239. doi: 10.1002/ctm2.1239 (PMC10111636; doi:10.1002/ctm2.1239)
Supplement: Supplementary file 11 — Supporting Information [file CTM2-13-e1239-s007.docx]

**Materials and Methods**

**RNAi, plasmid construction and cell transfection**

Small interfering RNAs (siRNAs) against target genes were purchased from GenePharma Corporation (Shanghai, China). The full-length sequence of BTG2 was cloned into the pcDNA3.1(+) vector (Invitrogen, Carlsbad, CA, USA). The full-length OGT sequence was cloned into pCMV-Myc-C (Invitrogen). The full-length sequence of LARP1, with or without mutated O-GlcNAcylated sites, was cloned into pcDNA3.1-3xFlag-C (Invitrogen). Flag-LARP1 deletion mutants were constructed as follows: for Flag-LARP1-Del-A plasmid, the LARP1 sequence encoding 356–1096 aa was cloned into Flag-tagged vector; for Flag-LARP1-Del-B plasmid, the LARP1 sequence encoding 516–1096 aa was cloned into Flag-tagged vector; for Flag-LARP1-Del-C plasmid, the LARP1 sequence encoding 813–1096 aa was cloned into Flag-tagged vector; for Flag-LARP1-Del-D plasmid, the LARP1 sequence encoding 1–813 aa was cloned into Flag-tagged vector. The full-length sequence of LARP1, with or without mutated ubiquitination sites, was cloned into pcDNA3.1-3xFlag-C (Invitrogen). All shRNAs and full-length sequences were cloned into pLVS-shRNA1 constructs (GenePharma). HB cells were transfected with siRNAs or plasmids using Lipofectamine 2000 (Invitrogen) in accordance with the manufacturer's protocol. The sequences of the constructs, shRNAs and siRNAs are listed in Table S3, S4 and S5.

**RNA immunoprecipitation (RIP) assay**

For RIP assays, the Magna RIP RNA-Binding Protein Immunoprecipitation Kit (Millipore, Billerica, MA, USA) was used following the manufacturer’s guidelines. Briefly, the cells were lysed using RIP lysis buffer plus RNase and protease inhibitors, and the supernatant was harvested. Primary antibodies against LARP1, FLAG, TRIM-25, or control IgG were incubated with protein A/G magnetic beads for 30 min at 37 °C. Afterwards, the supernatant was incubated with antibody-coated beads at 4 °C overnight. The co-precipitated RNA was pulled down using magnetic beads and analysed using qRT-PCR and RNA sequencing.

**Quantitative real-time RT-PCR (****qRT-PCR)**

Total RNA from tissues or cells was extracted using the TRIzol reagent (Invitrogen) and then reverse transcribed using the PrimeScript RT Reagent Kit with gDNA Eraser (Perfect Real Time) (Takara Bio, Kusatsu, Japan). The cDNA templates were subjected to quantitative real-time PCR analysis using TB Green Premix Ex Taq II (Tli RNaseH Plus) (Takara Bio). The 2^−ΔΔCT^ method was applied for the relative quantification of circRNAs and mRNAs using GAPDH to standardise RNA expression. Sequence of primers are listed in Table S6.

**Western blotting and co‑immunoprecipitation (co-IP) assay**

Total protein was extracted from tissues or cells using RIPA lysis buffer (Beyotime, Shanghai, China) containing protease and phosphatase inhibitors (Sangon Biotech, Shanghai, China) and used for western blotting as previously described [22]. Briefly, 40 μg of protein sample was separated by SDS-PAGE and then electrophoretically transferred to nitrocellulose membranes (GE Healthcare, Chicago, IL, USA). After blocking with BSA, these membranes were sequentially incubated with primary antibodies and IRDye 800CW Goat anti-Rabbit (or Mouse) IgG secondary antibodies (Listed in Table S7, used in accordance with product instructions). The bands on these membranes were visualised using an Odyssey infrared imaging system (LI-COR Biosciences).

For co-IP assays, HB cells were lysed with Pierce IP lysis buffer (Thermo Fisher Scientific, Waltham, MA, USA) containing protease and phosphatase inhibitors for 30 min on ice. After centrifugation, an aliquot of supernatant was harvested for protein expression analysis, and the remaining supernatant was precleared with Protein A/G Plus Agarose beads (Santa Cruz Biotechnology, CA, USA) for 1 h at 4 °C. IP was conducted with the addition of primary antibodies or control IgG (Millipore, NI01) and Protein A/G Plus Agarose beads. The mixtures were incubated with gentle rotation overnight at 4 °C. The beads were gently washed with IP lysis buffer five times, and the supernatant was collected for SDS-PAGE analysis.

**Immunohistochemistry (IHC) and immunofluorescence (IF) assay**

IHC assays were conducted using a tissue microarray consisting of 64 paired normal and HB tissues. Briefly, after deparaffinating, hydrating, retrieving antigen, blocking endogenous peroxidase, and non-specific staining, tissue microarrays were sequentially incubated with primary antibodies, biotinylated secondary antibodies, and peroxidase-labelled streptavidin, followed by incubation with the diaminobenzidine chromogenic substrate. Two specialised pathologists who were blinded to the clinicopathological information evaluated the staining intensities of the tissues at 100x and 400x magnification.

For immunofluorescence (IF) experiments, the HB cells were fixed with 4% paraformaldehyde solution, permeabilised with 0.5% Triton X-100 solution (Sigma, USA), and blocked with 5% bovine serum albumin. The cells were incubated with primary antibodies overnight at 4 °C, followed by incubation with secondary antibodies (Cell Signalling Technology) for 1 h at 37 °C. The cell nuclei were stained with 4,6-diamidino-2-phenylindole (Invitrogen). Finally, the slides were visualised under a Zeiss confocal fluorescence microscope.

**Cell proliferation assay**

To determine the proliferative activity of HB cells, they were seeded at 1000 cells/well in 96-well plates. After culturing for indicated days, 10 μl cell counting kit (CCK)-8 reagent (Beyotime Biotechnology, Shanghai, China) was added to each well, followed by incubation for 2 h at 37 °C. Absorbance at 450 nm was recorded using a Synergy 2 Multi-Mode Microplate Reader (Agilent Technologies, Winooski, VT, USA).

For colony formation assays, cells were seeded at 1000 cells/well in 12-well plates. After culturing for 8–10 days, the cells were washed, fixed, and stained with 500 μl 0.1% crystal violet for 0.5 h at room temperature. Finally, cell colonies were imaged and colony numbers were counted using ImageJ (U.S. National Institutes of Health, Bethesda, MD, USA).

**Cell apoptosis analysis**

The Annexin V-FITC Apoptosis Detection Kit (Beyotime Biotechnology) was used to evaluate HB cell apoptosis. Briefly, 2×10^5^ cells were harvested, washed with PBS, and resuspended in 195 μl Annexin V-FITC binding buffer. The cells were then stained with 5 μl Annexin V-FITC and 10 μl propidium iodide for 20 min in the dark at room temperature. Finally, the percentage of apoptotic cells was measured by FACS flow cytometry (BD Biosciences, San Jose, CA, USA).

**Transcriptome sequencing**

HuH6/shLARP1 and HuH6/shNC cells were subjected to mRNA sequencing using the Illumina NovaSeq 6000 platform (Illumina Inc., San Diego, CA, USA) in accordance with the manufacturer’s instructions. Sequencing data were then used to calculate the FPKM value of each gene with TopHat and Cufflinks software. The DESeq R package was used for differential expression analysis with a fold change (FC) >2 and P value < 0.05 as the threshold. The KEGG and the Database for Annotation, Visualization and Integrated Discovery were used to analyse signalling pathway enrichment and gene ontology, respectively. GSEA was used to identify the pivotal pathways associated with LARP1.
